# Impact of alcohol-induced intestinal microbiota dysbiosis in a rodent model of Alzheimer’s disease

**DOI:** 10.3389/fragi.2022.916336

**Published:** 2022-08-15

**Authors:** Dulce M. Frausto, Phillip A. Engen, Ankur Naqib, Aeja Jackson, Laura Tran, Stefan J. Green, Maliha Shaikh, Christopher B. Forsyth, Ali Keshavarzian, Robin M. Voigt

**Affiliations:** ^1^ Rush Center for Integrated Microbiome and Chronobiology Research, Rush University Medical Center, Chicago, IL, United States; ^2^ Genomics and Microbiome Core Facility, Rush University Medical Center, Chicago, IL, United States; ^3^ Department of Medicine, Rush University Medical Center, Chicago, IL, United States; ^4^ Department of Anatomy and Cell Biology, Rush University Medical Center, Chicago, IL, United States; ^5^ Department of Physiology, Rush University Medical Center, Chicago, IL, United States

**Keywords:** Alzheimer’s disease, microbiota, alcohol consumption, 3xTg-AD, behavior, brain pathology, intestinal barrier function

## Abstract

**Introduction:** Alzheimer’s disease (AD) is a devastating neurodegenerative disorder. While genetics are important in the development of AD, environment and lifestyle are also important factors influencing AD. One such lifestyle factor is alcohol consumption. Unhealthy and excessive chronic alcohol consumption is associated with a greater risk of all types of dementia, especially AD. Alcohol consumption has numerous effects on the body, including alterations to the intestinal microbiota (dysbiosis) and intestinal barrier dysfunction (leakiness and intestinal hyperpermeability), with evidence indicating that inflammation resulting from dysbiosis and barrier dysfunction can promote neuroinflammation impacting brain structure and function.

**Objective:** This study sought to determine the impact of alcohol-induced dysbiosis and barrier dysfunction on AD-like behavior and brain pathology using a transgenic rodent model of AD (3xTg-AD).

**Methods:** Alcohol (20%) was administered to 3xTg-AD mice in the drinking water for 20 weeks. Intestinal (stool) microbiota, intestinal barrier permeability, systemic inflammation (IL-6), behavior, and AD pathology (phosphorylated tau and β-amyloid), and microglia were examined.

**Results:** Alcohol consumption changed the intestinal microbiota community (dysbiosis) and increased intestinal barrier permeability in both control and 3xTg-AD mice (oral/urine sugar test and lipopolysaccharide-binding protein (LBP)). However, alcohol consumption did not influence serum IL-6, behavior, or β-amyloid, phosphorylated tau, or microglia in 3xTg-AD mice. Important differences in genotype and sex were noted.

**Conclusion:** Alcohol-induced microbiota dysbiosis and intestinal barrier dysfunction did not exacerbate behavior or AD-like brain pathology in the 3xTg-AD mouse model of AD which could, in part, be the result of a lack of systemic inflammation.

## Introduction

Currently, there are no therapies for Alzheimer’s disease (AD) that can prevent disease development or delay/halt disease progression. Thus, identifying environmental factors that promote AD are essential to develop new lifestyle recommendations and therapeutic approaches to prevent, delay, and slow the progression of AD. Epidemiological data provide compelling evidence that unhealthy alcohol consumption (particularly alcohol abuse and dependence) is associated with a higher than average risk of age-associated cognitive decline and AD (Sabia et al., 2014). Almost 80% of adults (>70 years of age) with a history of alcohol abuse have greater cognitive impairment and dementia compared to age-matched adults without a history of alcohol abuse (Thomas and Rockwood, 2001). While much of the literature shows that alcohol is detrimental for cognition, not all studies agree, which may stem from differences in the definitions of light, moderate and heavy drinking, or differences in approaches to identify AD (e.g., phone evaluation vs. comprehensive neurological exam) (Ruitenberg et al., 2002; Piazza-Gardner et al., 2013). Nonetheless, problematic drinking (e.g., binge alcohol use disorders) are consistently reported to negatively affect brain health in humans (Sullivan et al., 2010; Rao and Topiwala, 2020).

Studies in rodents similarly demonstrate that alcohol consumption detrimentally impacts cognitive function, learning, and memory and can alter levels of proteins characteristic of AD including tau, amyloid precursor protein (APP), and presenilin-1 (PSEN-1) in brain regions important for learning and memory, such as the CA1 region of the hippocampus and the basal lateral amygdala (BLA) (Laske et al., 2010; Huang et al., 2018; Hoffman et al., 2019). For example, administering alcohol to AD transgenic mouse models (3xTg-AD and APP/PSEN1) promotes AD-like pathology and behavior compared to non-alcohol–consuming AD transgenic mice (Huang et al., 2018; Hoffman et al., 2019; Gong et al., 2021). Taken together, there is ample evidence demonstrating that alcohol promotes AD-like neuropathology and cognitive deficits. However, the mechanism by which alcohol affects AD pathogenesis is still unclear but could include changes in the intestinal microbiota.

Pro-inflammatory changes in the intestinal microbiota are a well-documented consequence of alcohol consumption (Bode et al., 1984; Keshavarzian et al., 1999, 2009; Mutlu et al., 2009, 2012; Yan et al., 2011; Queipo-Ortuño et al., 2012; Bull-Otterson et al., 2013; Summa et al., 2013; Temko et al., 2017; Lee and Lee, 2021). These changes include a reduction in bacteria that are thought to be beneficial [e.g., Firmicutes (Engen et al., 2015) and Lachnospiraceae (Chen et al., 2011; Bull-Otterson et al., 2013)] with a concurrent increase in bacteria that are considered to be pro-inflammatory [e.g., Bacteroidetes (Yan et al., 2011; Queipo-Ortuño et al., 2012), Proteobacteria (Chen et al., 2011; Mutlu et al., 2012; Queipo-Ortuño et al., 2012), and Verrucomicrobia (Yan et al., 2011)]*.* The net result of these changes is a more pro-inflammatory microbiota profile.

It is well-established that the intestinal microbiota can impact the brain (neuroinflammation, function, structure, and behavior); therefore, alcohol-induced changes in the microbiota could influence cognition and risk of AD. The microbiota communicates with the brain *via* many mechanisms, and this study explored the relationship between alcohol-induced changes in the intestinal microbiota (and the intestinal barrier), peripheral inflammation, and the development/progression of AD-like outcomes (tau, β-amyloid, and microglia activation) in male and female 3xTg-AD mice.

## Methods and materials

### Animal model

All experiments were approved by the Rush University Institutional Animal Care and Use Committee (IACUC). AD triple-transgenic mice, B6; 129-Psen1^tm1Mpm^ Tg [amyloid precursor protein (APP)Swe, tauP301L]1Lfa/J (named 3xTg-AD), and their wild-type non-transgenic litter mates, B6129SF2 mice (named NonTg), were generated by Dr. Frank LaFerla at UC Irvine (Oddo et al., 2003) and provided for use in these studies *via* the Mutant Mouse Resource and Research Center Repository (MMRRC stock #34830) at Jackson Labs (Bar Harbor, ME) under an approved Material Transfer Agreement with UC Irvine.

A total of 108 mice, 54 3xTg-AD (29 female and 25 male) and 54 B6129SF2 (24 male and 30 female), were bred in house. All experiments were initiated when mice were 8 weeks old. Mice were fed Envigo 2018 standard rodent chow (Teklad, Madison, WI) with or without alcohol in the drinking water (described after). A standard 12 h light/12 h dark (6 a.m.–6 p.m.) schedule was maintained under specific pathogen-free conditions in isolator cages with 3–5 mice/cage. The general condition and health of the mice was monitored by daily observation and weekly body weight measurements.

### Treatment and timeline

**FIGURE 1:**
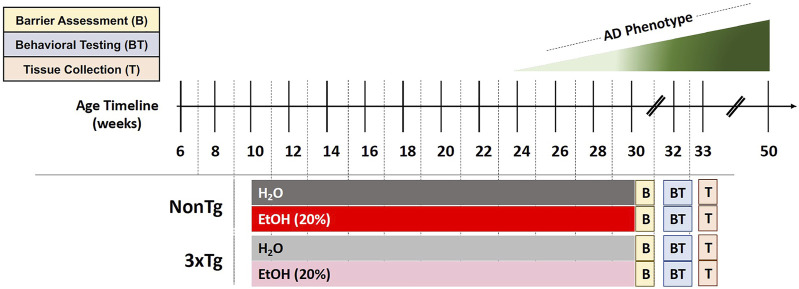
Experimental design. Behavioral and brain abnormalities begin to emerge around 24 weeks of age in 3xTg-AD mice with the most severe pathology observed at 50 weeks. EtOH (20%) or control (H_2_O) administration initiated when mice were 10 weeks of age and continued until 30 weeks of age. Barrier assessment (B) occurred at the end of the treatment. Behavioral testing (BT) occurred at 32–33 weeks of age followed by tissue collection (T) at 33 weeks of age.

Mice were administered with 20% alcohol (ethanol, EtOH) v/v treatment (or 0% EtOH water control) in the drinking water (replaced every other day), starting at 10 weeks until 30 weeks of age for a total duration of 20 weeks (Figure 1). Rodents metabolize alcohol more rapidly than humans (mice 5.5x greater than humans), and it is estimated that pharmacologically relevant blood alcohol levels (BAL) of approximately 1.0 g/L need to be achieved in rodents to be comparable to BAL in humans consuming alcohol (Jeanblanc et al., 2019). Administration of 20% ethanol (EtOH) in the drinking water to rodents leads to pharmacologically relevant BAL (>1.0 mg/ml) (Rhodes et al., 2005; Jeanblanc et al., 2019); thus, a dose of 20% EtOH was chosen for this study. Mice were acclimated to alcohol consumption *via* a 10-day ramp: 3% on days 1–2, 5% on days 3–4, 10% on days 5–7, 15% on days 8–9, and 20% on day 10. Control groups received drinking water from the same source but without alcohol. After 20 weeks, spontaneously voided stool samples were collected for assessment of microbial communities followed by a test for intestinal barrier integrity. Mice were then withdrawn from alcohol 2 weeks prior to behavioral testing to avoid potential confounding effects of alcohol intoxication on behavior. Finally, after behavioral analysis (33 weeks of age), mice were deeply anesthetized, blood was collected *via* cardiac puncture, animals were perfused with ice cold saline, and brain tissue was collected (Figure 1).

### Intestinal microbiota analysis

#### Stool collection, DNA extraction, and DNA sequencing

To collect stool for microbiota analysis, individual mice were placed into a cage without bedding for 12 h, after which spontaneously voided stool pellets were collected and stored at −80°C until analysis. Total genomic DNA was extracted from the stool pellets using the FastDNA SPIN Kit, according to manufacturer’s protocol (FastDNA SPIN Kit for soil, MP Biomedicals, Solon, OH), and quantified with fluorometric quantitation (Qubit 3.0, Life Technologies, Grand Island, NY, United States). To reduce batch effects, all samples were extracted using the same DNA extraction kit at the same time, and library preparation for all samples was conducted in 96-well plates simultaneously. Primers 515F/806R (515F: GTGTGYCAGCMGCCGCGGTAA; 806R: CCGGACTACNVGGGTWTCTAAT), modified from the Earth Microbiome Project primers and targeting the V4 variable region of microbial 16S ribosomal RNA (rRNA) genes, were used for PCR (Caporaso et al., 2012) and prepared for high-throughput amplicon sequencing using a two-stage PCR method, as previously described (Naqib et al., 2018). Sequencing was performed using an Illumina MiniSeq, with a V2 kit and paired-end 150 base reads at the Genomics and Microbiome Core Facility (GMCF) at Rush University Medical Center.

#### 16S rRNA V4 sequencing analysis

Raw sequences were merged using the software package PEAR (paired-end read merger) (v0.9.11) (Dalhousie University, Halifax, Nova Scotia, Canada) (Zhang et al., 2014). Merged sequences shorter than 240 bases were removed. Merged sequences were then processed (including denoising) using the DADA2 algorithm within the QIIME2 (v 2020.8.0) workflow (Callahan et al., 2016; Estaki et al., 2020). The amplicon sequence variants (ASVs) generated were used for all downstream analyses. Taxonomy was assigned to each ASV using the naïve Bayes classifier employing the SILVA 138 99% reference database (Quast et al., 2013; Bokulich et al., 2018). A total of 6,931,336 sequencing clusters were generated, with an average depth of 36,869 sequences per sample (median = 35,913; min = 0; max = 118,267). In total, six reagent contaminant ASVs (*Alistipes* (uncultured bacterium); *Pseudomonas*; *Clostridia* vadin BB60 group; *Clostridia* vadin BB60 group (uncultured bacterium); *Clostridia* UCG-014; and *Burkholderia–Caballeronia–Paraburkholderia*) were identified and removed using the decontam algorithm based on the prevalence of the ASVs in the reagent negative blank controls (*n* = 6) using default parameters (Davis et al., 2018). Unassigned and host-associated taxa, such as eukaryote, chloroplast, and mitochondrial ASVs, were removed from datasets prior to statistical analyses (Hanshew et al., 2013). Raw sequence data were deposited in the NCBI Sequence Read Archive under BioProject PRJNA781947.

### Intestinal barrier assessments

#### Urinary sugar test

*In vivo* intestinal barrier integrity was evaluated as previously described (Summa et al., 2013; Shaikh et al., 2015). In brief, mice were fasted for 8 hours prior to the test, which was performed at ZT0 (lights on). A 200 µl liquid solution containing lactulose (3.2 mg), sucrose (0.45 mg), sucralose (0.45 mg), and mannitol (0.9 mg) was given to mice *via* gavage, after which 2 ml of 0.9% saline was injected subcutaneously to promote urine output. Individual mice were placed in metabolic cages for 5 hours, after which urine was collected and the total volume was recorded. Intestinal barrier integrity was assessed by measuring urinary sugar concentration with gas chromatography and is expressed as percent excretion of oral dose of sugar (higher urinary sugar equates to greater barrier dysfunction) (Shaikh et al., 2015). Sucrose represents the barrier function in the stomach; duodenum, lactulose, and mannitol represent the small intestine (jejunum and ileum) (Müller et al., 1969; Ukabam and Cooper, 1984; Hodges et al., 1989; Bjarnason et al., 1994; Dumas et al., 1994; Johnston et al., 2000); and sucralose represents the whole intestine (Meddings et al., 1993).

#### Lipopolysaccharide-binding protein assay

Lipopolysaccharide (LPS) is a component in the outer membrane of Gram-negative bacteria, and LPS-binding protein (LBP) is a type 1 acute-phase protein that binds to LPS to facilitate an immune response and is a well-accepted marker of intestinal barrier integrity and endotoxemia. Serum collected at the time of euthanasia was used to measure LBP levels using an LBP ELISA kit (HK205; Hycult Biotech), according to the manufacturer’s instructions.

### Behavioral testing: Open field test

Spontaneous locomotor activity, habituation to a novel environment, and pivoting behavior of mice was measured in an open field chamber (i.e., a wooden floor square arena, 40 cm × 40 cm, with walls 30 cm high) (Seibenhener and Wooten, 2015). Mice were placed in the center of the arena and left to explore freely for 10 min. A video camera (Basler Gen I Cam with Basler acA 1300-60) connected to a Noldus computer system was placed above the box and recorded each session. The central area was defined as a square of 20 cm ×20 cm (half the total area). EthoVision XT software was used to analyze and store horizontal and vertical activity data, which were monitored automatically by infrared beams. Parameters analyzed included time spent in center chamber (s), velocity of movement (cm/s), total distance moved (cm), time spent immobile (freezing behavior), and body posture (normal and stretched elongation). These behaviors are thought to assess locomotor activity and anxiety-like behaviors (Carola et al., 2002; Seibenhener and Wooten, 2015), which are also observed in humans with AD. The OFT has been used to assess behavior in rodent models of AD (Hebda-Bauer et al., 2013).

### Tissue collection

Necropsy was performed under anesthesia (100 mg/kg ketamine and 20 mg/kg xylazine) as approved by Rush IACUC #19–079. Blood was collected *via* cardiac puncture and stored on ice until processing. Blood was spun at 2,000 RPM for 35 min at 22 ° C and serum was collected. After blood collection, mice were perfused with cold phosphate-buffered saline (PBS). The brain was collected and stored in paraformaldehyde (PFA) at 4°C overnight and then transferred to 30% sucrose until processing.

### Immunofluorescence brain analysis

Brain samples were cut at 40-μm thickness using a microtome [860, American Optical (AO)] and were stored in a cryoprotectant until use. Free-floating sections from 2.15 mm posterior to bregma were washed in PBS and then incubated in blocking solution (PBS with 0.25% Triton X-100, 1% bovine serum albumin (BSA), and 5% normal goat serum) for 1 h. Sections were incubated overnight at 4°C in the following primary antibodies diluted in blocking solution: β-amyloid 1–42 (1∶500, Bioss), phospho tau S404 (1∶500, Abcam), and Iba-1 (1:1,000, Abcam) followed by three consecutive 10 min washes in PBS. After washing, sections were incubated for 1 h at room temperature (22°C) in fluorescent secondary antibody (Alexa Fluor 488, 555, and 647, 1∶500, Abcam). Finally, sections were mounted onto slides and coverslipped in a Fluoroshield Mounting Medium already containing DAPI (ab104139, Abcam), according to the manufacturer’s instructions. Confocal images of hippocampus (CA1) and basal lateral amygdala (BLA) brain regions were acquired by sequential scanning using a Keyence BZ-X810 microscope at ×20 and ×40. Identical scan settings were used for all samples for each brain region analyzed.

Once collected, three images from the CA1 and BLA (*n* = 3 sections per mouse) were analyzed *via* ImageJ (Rasband, W.S., ImageJ, U. S. National Institutes of Health), and immunofluorescence of each antibody was quantified (mean difference between background and overall fluorescence of each region). Florescence for each image and section was averaged to determine the immunofluorescence value for each mouse (GraphPad Prism software).

### Blood alcohol levels

#### Sample preparation and gas chromatography–mass spectrometry analysis

Samples (25 µl each) were removed from storage (−80°C) and thawed at 4°C for 15 min. Internal standard (0.1% i.e., 0.804 mg/ml of n-propanol (nPrOH); 5 μL) was added to each sample. The sample material (pipette mixed) was transferred to a GC vial (2 ml) and capped immediately. The samples were put on the GC autosampler for a minimum 15 min at room temperature (22°–23°C) to allow liquid–vapor equilibrium prior to injection. The headspace vapor (25 μl) was injected (gastight 100 μl syringe) into a Trace 1310 GC coupled to a Thermo ISQ-LT MS with a split ratio of 10:1. The inlet was held at 250°C. Peak separation was achieved on a 30 m DB-WAX UI column (J and W, 0.25 mm ID, and 0.25 μm film thickness). The oven temperature was held at 40°C for 4 min and ramped at 40°C/min to 120°C, with a final hold for 1 min. The helium carrier gas flow was held at 1.2 ml/min. Temperatures of transfer line and ion source were both held at 250°C. The SIM mode was used to scan ions m/z 31 and 45 for EtOH, m/z 31 and 42 for nPrOH, and m/z 59 and 43 for iPrOH (IPA) with a scan time of 0.1 s/ion under the electron impact mode.

#### Data analysis

GC/MS data were analyzed with Chromeleon. Each target analyte (EtOH, nPrOH, and iPrOH) was visually inspected for retention time and peak area integration. Peak areas for EtOH and nPrOH were extracted for each sample. Absolute quantitation (mg/mL and %) was calculated using the linear regression equation generated for each compound from the calibration curve.

### Serum interleukin-6 analysis

Serum cytokine interleukin-6 (IL-6) levels were assessed with a Meso Scale V-PLEX Pro-inflammatory Mouse Kit (Cat. #K152QXG-1, Meso Scale Diagnostics, Rockville, MD). The samples were analyzed in duplicate, and assays were performed on a QuickPlex SQ120 (Meso Scale Diagnostics), according to the manufacturer’s protocol.

### Statistical analysis

#### Tissue and behavioral statistical analyses

Data are reported as mean ± standard error of the mean (SEM). A two-way analysis of variance (ANOVA) was used to evaluate the main effects of genotype (NonTg vs. 3xTg-AD), treatment (H_2_O vs. EtOH), and interaction. Planned comparisons (*a priori*) were conducted between groups using a Tukey test (to reduce type I error) (Ruxton and Beauchamp, 2008). Weight differences were analyzed using a repeated measures three-way ANOVA to assess the main effects of genotype (NonTg vs. 3xTg-AD), treatment (H_2_O vs. EtOH), time (week), and interactions. Differences in sex were assessed using a two-way ANOVA to evaluate the main effects of sex (female vs. male), treatment (H_2_O vs. EtOH), and interaction. Significance was set at *p* < 0.05. Analyses were conducted using GraphPad Prism (v9.1) software (GraphPad Software, La Jolla, CA).

Spearman’s correlations were used to assess the relationship between relative abundances of specific taxa (species) and AD-like phenotype and to identify potential targets for future studies. Significant threshold of *p*-value was set at *p* < 0.05 and R > 0.30 (R Project, 2022). This approach has been used previously to identify a relationship between specific bacterial species with AD-like outcomes (Vogt et al., 2017).

#### Microbiota analysis

Analyses of alpha and beta diversity were used to compare the stool microbial community structure between groups (e.g., male vs. female; EtOH vs. H_2_O; 3xTg-AD vs. NonTg). All analyses were performed on feature (ASV) counts. Alpha diversity metrics [i.e., Shannon index, Simpson’s index, Observed Features (number of taxa), and Pielou’s Evenness (relative abundance of those taxa)] were calculated on rarefied datasets (6,500 sequences/sample). Significance was considered at *p* < 0.05. These analyses were performed using the software package GraphPad Prism (v9.1, GraphPad Software LLC, San Diego, California).

Permutation multivariate analysis of variance (PERMANOVA) based on the Bray–Curtis distance matrix was used to assess global differences in microbial communities between mice groups (Kelly et al., 2015). Significance was determined using 9,999 permutations, and adjustment for multiple testing was conducted using the Benjamini–Hochberg FDR correction. Visualization of data was performed using the principal coordinates analysis (PCoA) based on a Bray–Curtis dissimilarity distance matrix within the software package QIIME2 (Estaki et al., 2020). Differential abundance analyses of individual taxa between groups were performed using the software package DESeq2, generating an FDR *q*-value (Love et al., 2014; Li and Andrade, 2017). DESeq2 has been shown to be appropriate for differential abundance comparisons in studies with small sample size groups (˂20), or unbalanced design (Weiss et al., 2017). Individual taxa percent mean relative abundances (˃1%) ± standard deviations (SD) were calculated and depicted as stacked histograms.

## Results

### Sex differences: Microbiota

One of the primary goals was to understand the impact of alcohol-induced changes in the intestinal microbiota on the AD-like phenotype. Sex differences in microbiota have been documented in both human and animal studies; therefore, between sex analysis of microbiota was conducted to determine if males and females could be pooled for subsequent analyses (Fushuku and Fukuda, 2008; Markle et al., 2013; Yurkovetskiy et al., 2013; Org et al., 2016; Fransen et al., 2017; Elderman et al., 2018; Kim et al., 2020). Analysis of stool microbial communities revealed that both alpha diversity (Supplementary Table S1) and stool microbial community structures (PERMANOVA *q* ˂ 0.02) were significantly different between male and female mice. Based on this outcome, all subsequent analyses examined males and females separately.

### Impact of alcohol consumption in female mice

#### Chronic alcohol consumption: Blood alcohol and weight gain in female mice

The blood alcohol levels were assessed, but no significant effects were noted, which is consistent with blood collected following a 2 week alcohol withdrawal period (data not shown). However, alcohol consumption is sometimes associated with reduced weight gain. While neither genotype nor treatment were significant, time was a significant factor (*p* < 0.01), and there was a significant treatment × genotype × time interaction (*p* < 0.01) (Supplementary Figure S1A). In additional, the changes in the intestinal milieu of alcohol-fed mice are consistent with changes previously observed in alcohol-fed rodents.

#### Chronic alcohol consumption is associated with altered intestinal microbiota in female mice

Intestinal microbiota alterations are observed in AD mouse models, including 3xTg-AD (Bello-Medina et al., 2021); therefore, we first evaluated the impact of genotype on stool microbial communities (i.e., NonTg-H_2_O + NonTg-EtOH vs. 3xTg-AD-H_2_O + 3xTg-AD-EtOH). No significant differences in alpha diversity were observed (i.e., variation within each sample) (data not shown); however, analysis of beta diversity revealed a significant impact of genotype on the microbiota (i.e., differences between samples/groups) (Supplementary Figure S2A; Table 1). 3xTg-AD mice had a significantly different microbiota profile compared to the NonTg mice, which included a concurrent increase in the relative abundance of genera reported to be changed in rodent models and humans with neurodegenerative disease including *Faecalibaculum* (*q* < 0.05) and less stringent (defined as *q* > 0.05; *p* < 0.05) genera *Bifidobacterium* and Peptococcaceae (genus uncultured) that did not meet the *q*-value standard (Table 2). Additional putatively pro-inflammatory genera were noted as increased in 3xTg-AD compared to NonTg mice, which included *Muribaculaceae* and *Parasutterella* (both *q* < 0.05) and less stringent genera *Alloprevotella* (*q* > 0.05; *p* < 0.05), as well as genera not previously mentioned in neurodegeneration or alcohol literature including *Candidatus Stoquefichus*, Atopobiaceae (genus unknown), *Erysipelatoclostridium*, Butyricicoccaceae UCG-009, and *Anaerotruncus* (all *q* < 0.05) (Table 2). Last, 3xTg-AD mice had a lower average relative abundance of putatively beneficial short-chain fatty acids (SCFA)–producing genera Lachnospiraceae NK4B4 group, as well as other genera such as Anaerovoracaceae, (*Eubacterium*) *brachy* group, *Staphylococcus*, and *Akkermansia* (all *q* < 0.05) (Table 2). Taken together, these data indicate that microbiota dysbiosis is present in 3xTg-AD female mice compared to the NonTg mice.

**TABLE 1:** Permutational multivariate analysis of variance (PERMANOVA). PERMANOVA results are based on the Bray–Curtis distance matrix. Significance was determined using 9,999 permutations and corrected or multiple testing using the Benjamini–Hochberg method (*q* < 0.05, indicated by bold). Groups include NonTg H_2_O-fed (*n* = 10); NonTg EtOH-fed (*n* = 10); 3xTg-AD H_2_O-fed (*n* = 10); and 3xTg-AD EtOH-fed (*n* = 10), per sex.

Comparison	Feature taxonomic level		
	Sample size (per group)	Psuedo-F	q-value
Females (All) vs. males (All)	40	2.298	**< 0.01**
Females			
NonTg vs. 3xTg-AD	20	4.138	**< 0.01**
H_2_O vs. EtOH	20	2.588	**< 0.01**
NonTg: H_2_O vs. EtOH	10	1.615	0.07
3xTg-AD:H_2_O vs. EtOH	10	3.240	**< 0.01**
Males			
NonTg vs. 3xTg-AD	20	3.996	**< 0.01**
H_2_O vs. EtOH	20	2.916	**< 0.01**
NonTg: H_2_O vs. EtOH	10	2.500	**< 0.01**
3xTg-AD:H_2_O vs. EtOH	10	2.436	**< 0.01**

**TABLE 2:** DeSeq2—NonTg vs. 3xTg-AD—females. DeSeq2 Analysis. Taxa shown have adjusted *p*-values (*p*-value < 0.05 indicated by italics; *q*-value < 0.05 indicated by bold). Base mean = mean of normalized samples. Log2 FC = Log2 fold change of taxa in 3xTg-AD mice compared to NonTg mice within the respective genotype.

DeSeq2—NonTg vs. 3xTg-AD—females				
Genera (phylum)	Base mean	Log2 FC 3xTg-AD over NonTg	*p*-value	q-value
NonTg (H_2_O + EtOH, *n* = 20) vs. 3xTg-AD (H_2_O + EtOH, *n* = 20)				
Neurodegenerative disease related (*)				
*Faecalibaculum* (Firmicutes)	1028.68	5.01	*< 0.01*	**< 0.01**
*Bifidobacterium* (Actinobacteriota)	896.02	2.45	*0.01*	0.10
Peptococcaceae (genus uncultured) (Firmicutes)	98.40	1.59	*< 0.01*	**< 0.01**
Putatively pro-inflammatory (**)				
*Parasutterella* (Proteobacteria)	136.62	2.71	*<0.01*	**0.02**
*Alloprevotella* (Bacteroidota)	3220.18	1.78	*0.02*	0.12
*Muribaculaceae* (Bacteroidota)	14183.22	0.88	*< 0.01*	**< 0.01**
Putatively beneficial				
*Lachnospiraceae* (NK4B4 group) (Firmicutes)	2.93	−3.69	*< 0.01*	**0.01**
Additional genera				
*Candidatus stoquefichus* (Firmicutes)	33.26	5.58	*< 0.01*	**< 0.01**
Atopobiaceae (genus unknown) (Actinobacteriota)	78.89	3.06	*< 0.01*	**0.02**
*Erysipelatoclostridium* (Firmicutes)	81.09	2.98	*< 0.01*	**< 0.01**
*Butyricicoccaceae* UCG-009 (Firmicutes)	8.81	2.25	*< 0.01*	**0.02**
*Peptococcus* (Firmicutes)	3.76	2.08	*0.02*	0.12
*Anaerotruncus* (Firmicutes)	22.25	1.78	*< 0.01*	**0.03**
Anaerovoracaceae*;(Eubacterium) brachy group* (Firmicutes)	13.66	−1.21	*< 0.01*	**0.03**
*Streptococcus* (Firmicutes)	16.30	−1.83	*0.03*	0.14
*Anaeroplasma* (Firmicutes)	23.61	−1.87	*0.03*	0.18
*Oscillospirales* (genus unknown) (Firmicutes)	2.91	−2.00	*0.04*	0.18
*Anaerofustis* (Firmicutes)	0.62	−2.08	*0.05*	0.23
*Staphylococcus* (Firmicutes)	4.75	−2.94	*0.01*	**< 0.05**
*Akkermansia* (Verrucomicrobiota)	323.56	−3.33	*< 0.01*	**0.02**

Stool microbial communities were examined for an alcohol treatment effect (i.e., NonTg-H_2_O + 3xTg-AD-H_2_O vs. NonTg-EtOH + 3xTg-AD-EtOH). No significant differences in alpha diversity were observed (data not shown); however, beta diversity was significantly impacted by alcohol consumption (Supplementary Figure S2B; Table 1). Alcohol consumption was associated with a dysbiotic microbiota profile, recapitulating previous studies demonstrating chronic alcohol consumption effects on the microbiota (rodent models and humans). Specifically, an increase in the relative abundance of Muribaculaceae and *Clostridium sensu stricto 1* (all *q* < 0.05), with less stringent genera *Faecalibaculum*, Anaerovoracaceae (genus unclassified), *Parasutterella*, *Blautia*, Bacilli RF39, and *Enterorhabdus* (all *q* > 0.05; *p* < 0.05), as well as additional genera not previously mentioned in alcohol-related literature Defluviitaleaceae and Christensenellaceae R-7 group (all *q* < 0.05) (Table 3). Last, alcohol consumption was associated with a reduction of the relative abundance of putatively beneficial SCFA-producing genera including *Lachnospiraceae* A2 and *Lachnospiraceae* GCA-900066575 (all *q* < 0.05) (Table 3). Collectively, these microbiota alterations are consistent with alcohol-induced changes reported in the literature.

**TABLE 3:** DeSeq2—H_2_O vs. EtOH—females. DeSeq2 analysis. Adjusted *p*-values (*p*-value < 0.05 indicated by italics; *q*-value < 0.05 indicated by bold). Base mean = mean of normalized samples. Log2 FC = Log2 fold change of taxa in EtOH-fed mice compared to H_2_O-fed mice samples within the respective genotype.

DeSeq2—H_2_O vs. EtOH—females				
Genera (phylum)	Base mean	Log2 FC EtOH over H_2_O	*p*-value	q-value
H_2_O (NonTg + 3xTg-AD, *n* = 20) vs. EtOH (NonTg + 3xTg-AD, *n* = 20)				
Alcohol consumption related (*)				
*Clostridium sensu stricto 1* (Firmicutes)	395.40	2.92	*< 0.01*	**0.04**
*Muribaculaceae* (Bacteroidota)	14183.22	0.87	*< 0.01*	**0.01**
*Faecalibaculum* (Firmicutes)	1060.75	2.68	*0.01*	0.10
Anaerovoracaceae (genus unknown) (Firmicutes)	2.05	2.37	*0.02*	0.20
*Parasutterella* (Proteobacteria)	136.62	1.71	*0.05*	0.26
Peptococcaceae (genus uncultured) (Firmicutes)	98.40	0.89	*0.03*	0.21
Bacilli RF39 (Firmicutes)	122.82	0.86	*0.04*	0.26
*Enterorhabdus* (Actinobacteriota)	550.65	0.79	*0.03*	0.21
Putative beneficial				
*Lachnospiraceae* GCA-900066575 (Firmicutes)	76.20	−1.35	*< 0.01*	0.09
*Lachnospiraceae* A2 (Firmicutes)	105.84	−1.70	*< 0.01*	0.05
Additional genera				
*Defluviitaleaceae* UCG-011 (Firmicutes)	2.91	4.08	*< 0.01*	**0.01**
*Christensenellaceae R-7 group* (Firmicutes)	5.41	3.03	*< 0.01*	**0.01**
Rhodospirillales (genus uncultured) (Proteobacteria)	2.51	2.85	*0.01*	0.16
Muribaculaceae (genus unknown) (Bacteroidota)	102.45	2.72	*0.01*	0.12
*Coriobacteriaceae* UCG-002 (Actinobacteriota)	26.09	2.33	*0.03*	0.21
Ruminococcaceae; (*Eubacterium*) *siraeum group* (Firmicutes)	46.36	2.05	*0.03*	0.21
Oscillospirales (genus unknown) (Firmicutes)	2.91	1.91	*0.05*	0.26
*Oscillospirales* UCG-010 (Firmicutes)	17.51	1.45	*0.01*	0.16
Oscillospiraceae (genus uncultured) (Firmicutes)	235.55	0.68	*0.05*	0.26
Anaerovoracaceae; (*Eubacterium*) *nodatum group* (Firmicutes)	16.33	−1.01	*0.04*	0.26
*Ruminococcaceae* (genus unknown) (Firmicutes)	10.74	−1.91	*0.03*	0.22
NonTg: H_2_O (*n* = 10) vs EtOH (*n* = 10)				
Alcohol consumption related				
Anaerovoracaceae (genus unclassified) (Firmicutes)	2.05	2.93	*0.03*	0.71
Putatively beneficial				
*Lachnospiraceae* A2 (Firmicutes)	105.84	−2.06	*0.01*	0.48
Additional genera				
*Clostridia* UCG-014 (Firmicutes)	406.77	−1.17	*0.04*	0.71
*Parvibacter* (Actinobacteriota)	37.55	−1.75	*0.04*	0.71
*Peptococcus* (Firmicutes)	3.76	−3.49	*0.01*	0.48
3xTg-AD:H_2_O (n = 10) vs. EtOH (n = 10)				
Alcohol consumption related (*)				
*Coriobacteriaceae* UCG-002 (Actinobacteriota)	171.46	5.84	*< 0.01*	**< 0.01**
*Ruminococcus* (Firmicutes)	155.81	3.72	*< 0.01*	**< 0.01**
*Clostridium sensu stricto 1* (Firmicutes)	395.4	3.70	*< 0.01*	**0.03**
Bacilli RF39 (Firmicutes)	122.82	1.43	*0.02*	0.10
Peptococcaceae (genus uncultured) (Firmicutes)	98.4	1.27	*0.02*	0.13
Oscillospiraceae (genus uncultured) (Firmicutes)	235.55	1.25	*0.01*	0.06
*Muribaculaceae* (Bacteroidota)	14183.22	1.13	*< 0.01*	**< 0.01**
Putatively beneficial				
*Lachnospiraceae* GCA-900066575 (Firmicutes)	76.2	−1.99	*< 0.01*	**0.03**
Additional genera				
*Candidatus Saccharimonas* (Patescibacteria)	21.7	5.62	*< 0.01*	**< 0.01**
*Christensenellaceae R-7 group* (Firmicutes)	5.41	5.32	*< 0.01*	**< 0.01**
*Oscillospiraceae* UCG-005 (Firmicutes)	9.84	3.91	*< 0.01*	**< 0.01**
*Defluviitaleaceae* UCG-011 (Firmicutes)	2.91	3.52	*0.02*	0.10
Ruminococcaceae*; [Eubacterium] siraeum group* (Firmicutes)	46.36	3.04	*0.02*	0.10
Muribaculaceae (genus unclassified) (Bacteroidota)	102.45	2.92	*0.04*	0.17
*Monoglobus* (Firmicutes)	41.18	2.64	*0.01*	0.07
*Oscillospirales* UCG-010 (Firmicutes)	17.51	2.09	*0.01*	0.08
*Clostridia* UCG-014 (Firmicutes)	406.77	2.02	*< 0.01*	**< 0.01**
*Clostridia* vadin BB60 group (Firmicutes)	87.35	1.78	*< 0.01*	**< 0.01**
*Intestinimonas* (Firmicutes)	35.21	−1.37	*< 0.01*	**0.03**

Next, microbial communities were examined for treatment effects within each genotype (i.e., NonTg: H_2_O vs. EtOH and 3xTg-AD:H_2_O vs. EtOH). There were no significant between group differences noted for alpha diversity (i.e., variation within each sample) (data not shown) or beta diversity between NonTg water- and alcohol-consuming mice (i.e., differences between samples/groups) (Supplementary Figure S2C; Table 1). No between-group differences were noted using the stringent *q*-value standard of significance. However, some bacteria were different based on a less stringent *p*-value threshold including increased relative abundance of putatively alcohol consumption–associated taxa Anaerovoracaceae (genus unclassified) with a lower average relative abundance of putatively beneficial SCFA-producing genus *Lachnospiraceae* A2 (all *p* < 0.05) (Table 3).

Alcohol-associated changes in the microbiota of 3xTg-AD mice were more robust than those observed in NonTg mice. Alpha diversity was not different between 3xTg-AD control and alcohol-consuming mice (data not shown), but there was a significant group difference in beta diversity (Supplementary Figure S2D; Table 1). Specifically, alcohol consumption increased the abundance of chronic alcohol consumption–associated genera Coriobacteriaceae, Muribaculaceae, *Ruminococcus*, and *Clostridium sensu stricto 1* (all *q* < 0.05), as well as less stringent Bacilli RF39, Peptococcaeceae (genus uncultured), and Oscillospiraceae (genus uncultured) (all *q* > 0.05; *p* < 0.05), and additional genera (not currently associated with alcohol consumption in the literature) such as *Candidatus Saccharimonas*, Christensenellaceae R-7 group, Oscillospiraceae UCG-005, *Clostridia* UCG-014, and *Clostridia* vadin BB60 group (all *q* < 0.05) (Table 3)*.* These changes were accompanied by a concurrent decrease in the relative abundance of bacteria from the genus *Intestinimonas* and putatively beneficial *Lachnospiraceae* GCA-900066575 (all *q* < 0.05) (Table 3).

Taken together, the richness (alpha diversity) of the intestinal microbiota was not impacted by genotype or treatment; however, microbial communities from samples within each treatment group were more similar to each other as compared to microbial communities in other groups (beta diversity). We demonstrated that 3xTg-AD female mice have a different microbiota composition than NonTg female mice (genotype effect) and that alcohol alters the microbial communities in 3xTg-AD and NonTg female mice (alcohol treatment effect).

#### Chronic alcohol consumption is associated with disrupted intestinal barrier in female mice

**FIGURE 2:**
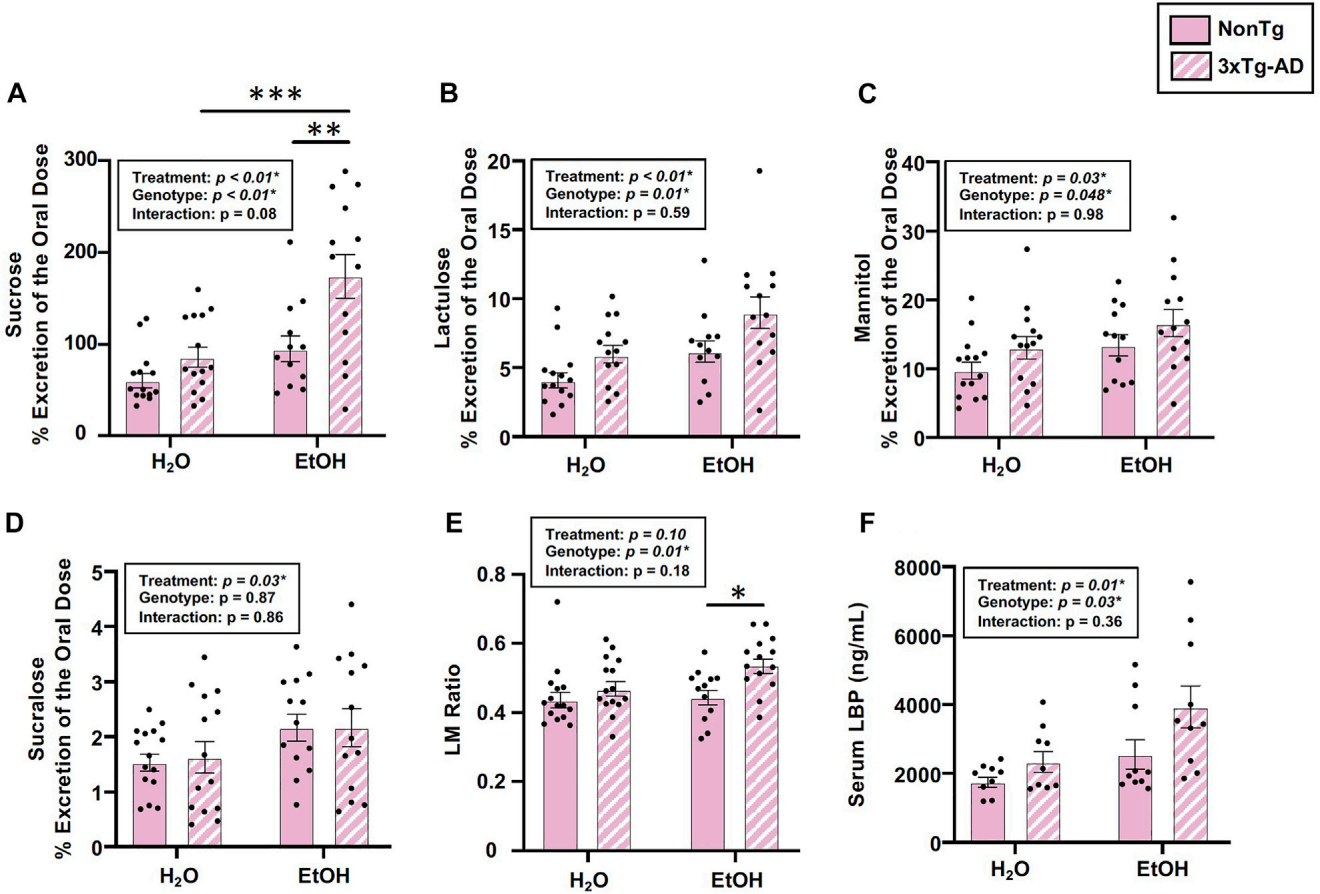
Effect of alcohol consumption on markers of intestinal barrier integrity in female mice. **(A)** Urinary sucrose exhibited a significant effect of genotype and alcohol treatment but no interaction. **(B)** Urinary lactulose exhibited a significant effect of genotype and alcohol treatment but no interaction. **(C)** Urinary mannitol exhibited a significant effect of genotype and alcohol treatment but no interaction. **(D)** Urinary sucralose was significantly impacted by alcohol treatment, but there was no effect of genotype nor was there an interaction. **(E)** Lactulose:mannitol (LM) ratio was significantly impacted by the genotype, but there was no effect of alcohol treatment nor was there an interaction. **(F)** Serum LBP exhibited a significant effect of genotype and alcohol treatment but no interaction. Between *n* = 6–14 mice/treatment group. Two-way ANOVA (results in box) was followed by planned (i.e., *a priori*) between-group comparisons, which are indicated on each graph when significant: ^∗^ *p* < 0.05, ^∗∗^ *p* < 0.01, and ^∗∗∗^ *p* < 0.001.

Analysis of urinary sugar content revealed a significant effect of genotype on the intestinal barrier for sucrose, lactulose, mannitol, and the lactulose:mannitol (LM) ratio, but the genotype did not impact sucralose (Figures 2A–E). Between-group testing indicated urinary sucrose and the LM ratio was higher in alcohol-fed 3xTg-AD mice compared to alcohol-fed NonTg mice (Figures 2A,E). An analysis of LBP (collected 2 weeks after alcohol treatment, see Figure 1) indicated a significant effect of genotype on LBP levels consistent with 3xTg-AD mice having disrupted intestinal barrier integrity compared to NonTg mice (Figure 2F).

Next, we examined the impact of alcohol treatment on intestinal barrier integrity. As expected, alcohol consumption (i.e., treatment) significantly impacted intestinal barrier integrity including urinary sucrose, lactulose, mannitol, and sucralose, but it did not impact the LM ratio (Figures 2A–E). Between-group testing revealed significantly higher levels of urinary sucrose in alcohol-consuming 3xTg-AD mice compared to the water-fed 3xTg-AD mice (Figure 2A). An analysis of LBP levels also demonstrated a significant effect of alcohol treatment (Figure 2F). Taken together, alcohol robustly impacted intestinal barrier integrity. No interaction effects were noted in any outcome assessed. These data are summarized in Supplementary Table S2.

#### Alcohol consumption did not alter AD-relevant behavior or brain pathology in female mice

**FIGURE 3:**
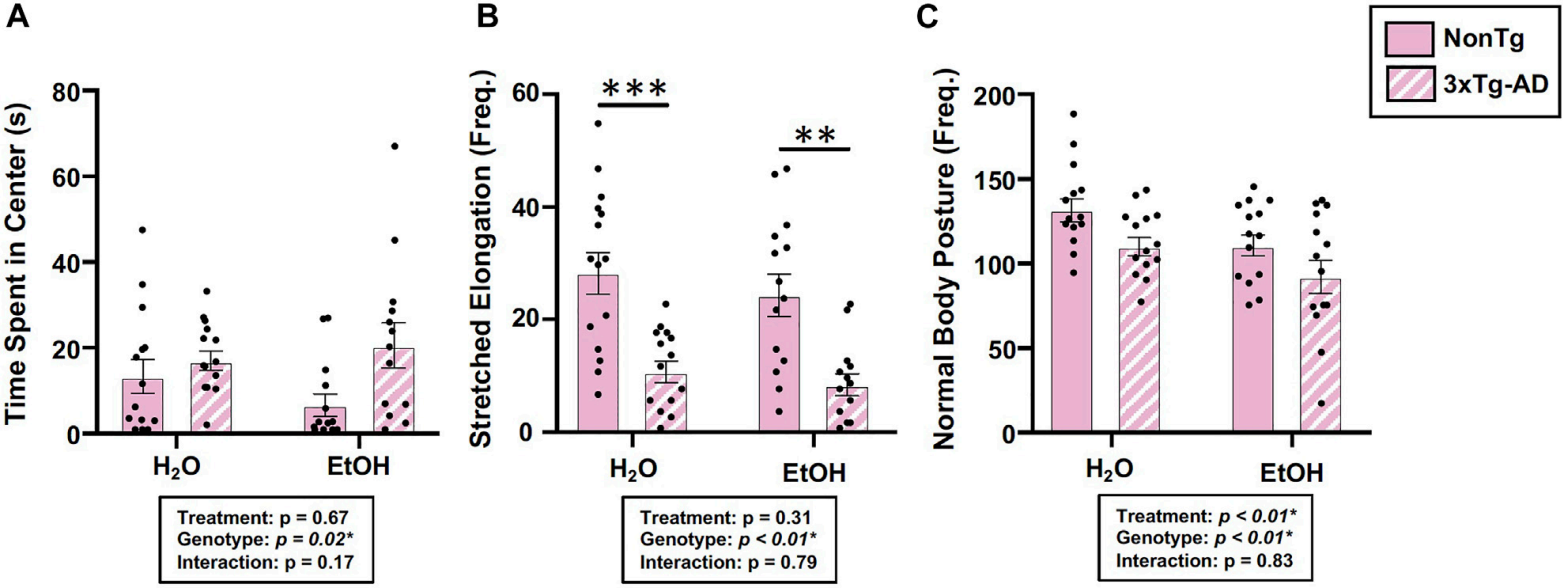
Effect of alcohol consumption on behavior in female mice. **(A)** Time spent in center was impacted by the genotype but not by alcohol consumption nor was there an interaction. **(B)** Frequency of stretched elongation body posture was impacted by the genotype but not by alcohol consumption nor was there an interaction. **(C)** Frequency of normal body posture exhibited a significant effect of genotype and treatment but not a significant interaction. Between *n* = 6–14 mice/treatment group. Two-way ANOVA (results in box) was followed by planned (i.e., *a priori*) between group comparisons, which are indicated on each graph when significant: ^∗^^∗^ *p* < 0.01 and ^∗^^∗^^∗^ *p* < 0.001.

Behavior was assessed using the open field test (OFT), and an analysis revealed a significant main effect of genotype on time spent in center, frequency of stretched elongation posture, and frequency of normal body posture (Figures 3A–C) but no effect on total distance moved, velocity of movement, or time spent immobile (data not shown). Between-group testing showed that stretched body elongation was significantly less frequent in 3xTg-AD mice compared to the NonTg mice for both H_2_O and EtOH treatment (Figure 3B). Alcohol treatment was a significant factor only for frequency of normal body posture (Figure 3C).

**FIGURE 4:**
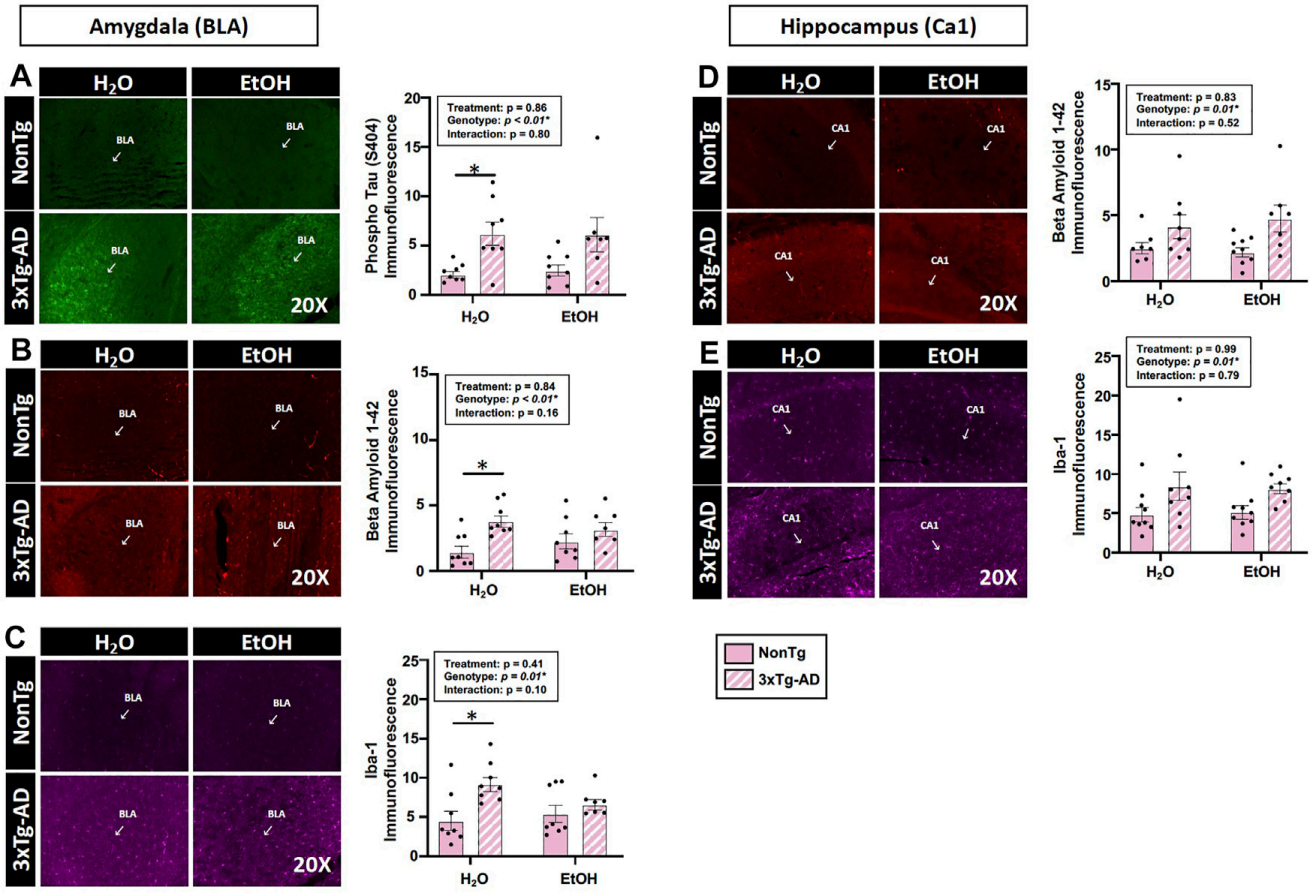
Effect of alcohol consumption of AD-like brain pathology in female mice. **(A–C)** BLA region of the brain. **(A)** Phosphorylated tau exhibited a significant effect of genotype but no effects of alcohol treatment nor was there an interaction. **(B)** β-amyloid exhibited a significant effect of genotype but was not impacted by alcohol consumption nor was there an interaction. **(C)** Iba-1 exhibited a significant genotype effect but was not impacted by alcohol consumption nor was there an interaction. **(D–E)** Hippocampus (CA1). **(D)** β-amyloid was impacted by the genotype but was not significantly impacted by chronic alcohol consumption nor was there an interaction. **(E)** Iba-1 exhibited a significant effect of the genotype but no effect of alcohol consumption nor was there an interaction. Between *n* = 6–14 mice were included in each treatment group. Two-way ANOVA (results in box) was followed by planned (i.e., *a priori*) between-group comparisons, which are indicated on each graph when significant: ^∗^ *p* < 0.05.

Assessments of brain pathology indicated a significant effect of genotype. In the BLA, there was a significant main effect of genotype on phospho tau, β-amyloid, and Iba-1 (Figures 4A–C). Between-group testing revealed that pathology was significantly higher in the water-fed 3xTg-AD compared to water-fed NonTg mice for all outcomes assessed in the BLA (Figures 4A–C). In the hippocampus, there was a significant main effect of genotype on β-amyloid and Iba-1 (Figures 4A–C) but no effect on phospho tau (data not shown). No significant effects of treatment were observed on any outcome assessed nor were there any significant interactions. A summary of these data is found in Supplementary Table S2.

#### Alcohol consumption did not alter peripheral inflammation in female mice

**FIGURE 5:**
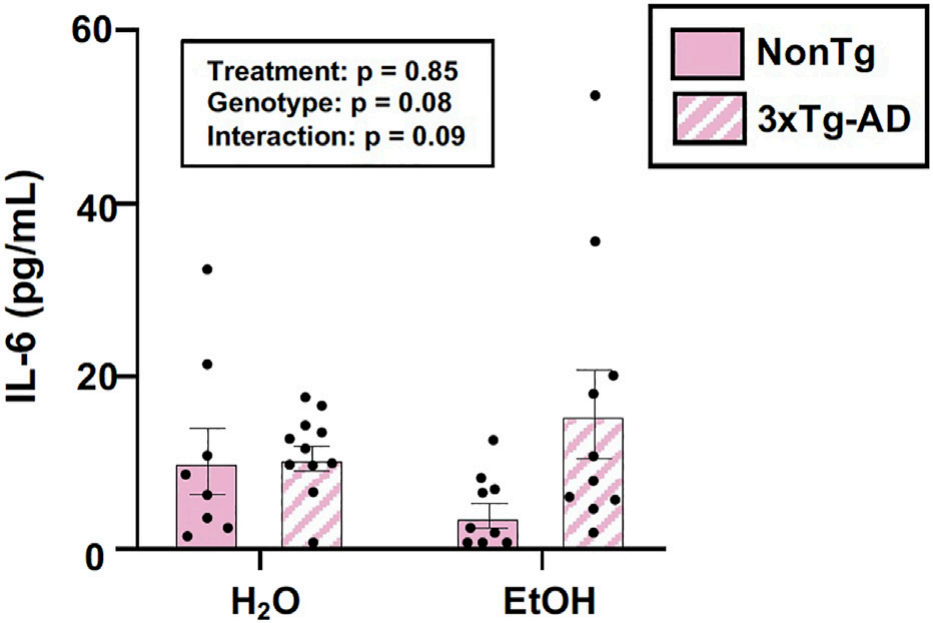
Effect of alcohol consumption on peripheral inflammation in female mice. Serum cytokine IL-6 levels were measured and analyzed. IL-6 (pg/ml) was not impacted by the genotype or alcohol consumption nor was there an interaction. Between *n* = 6–14 mice/treatment group. Two-way ANOVA (results in box).

The intestinal microbiota and the intestinal barrier robustly influence the immune system and inflammation. This is important as inflammation is proposed as one important mechanism by which the microbiota (and the barrier) communicates with the brain. Therefore, serum IL-6 levels were examined. An analysis revealed no effects of genotype, treatment, nor was there an interaction (Figure 5). The lack of peripheral inflammation could be a reason why the changes in the intestine did not potentiate the AD-like phenotype in 3xTg-AD mice. A summary of these data are found in Supplementary Table S2.

#### Relationship between the microbiota and AD-relevant outcomes in 3xTg-AD female mice

**FIGURE 6:**
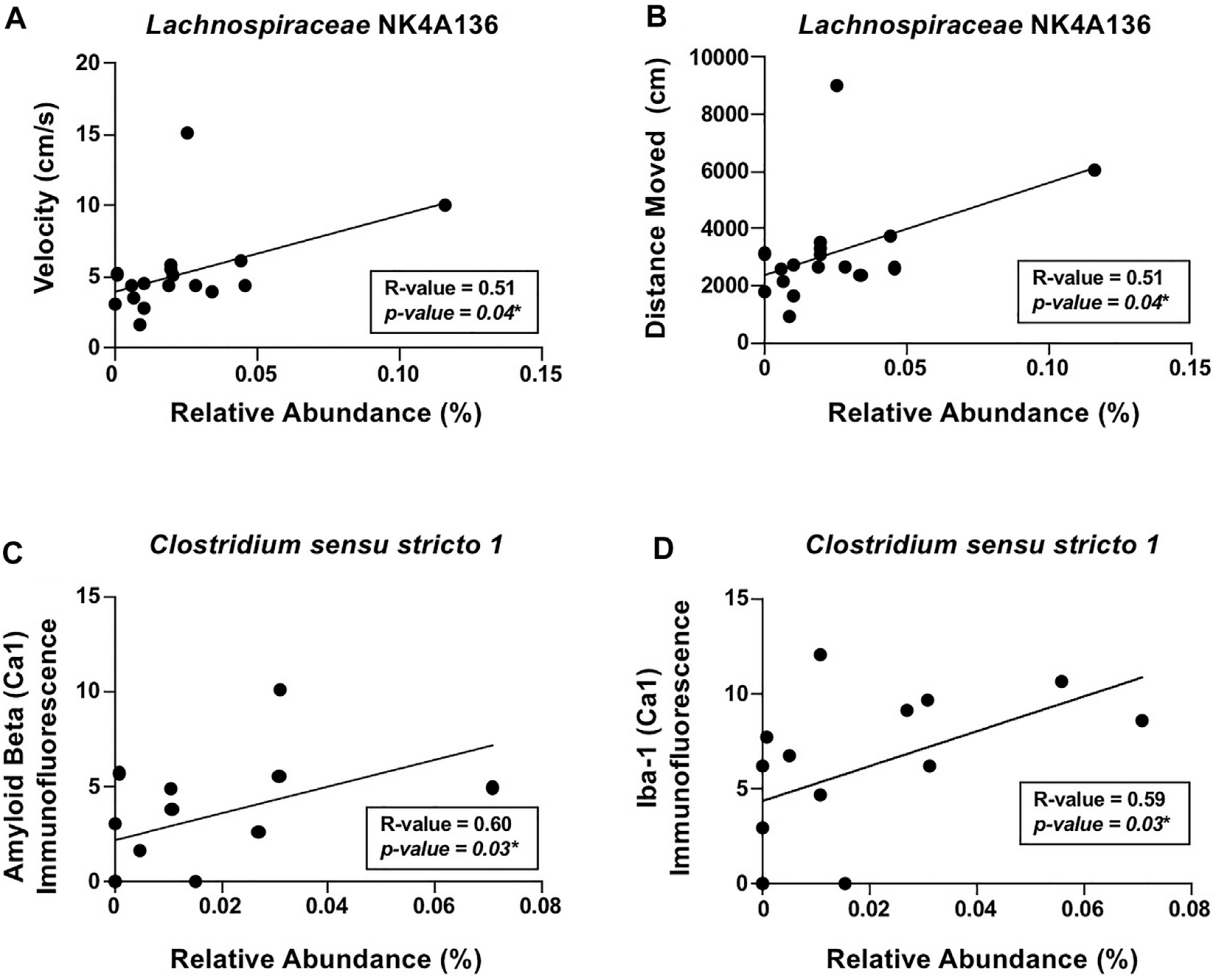
Relationship between the intestinal microbiota and AD-like behavior and brain pathology in female mice. Significant correlations were noted between **(A)** velocity of movement (cm/s) and *Lachnospiraceae* NK4A136, **(B)** distance moved (cm) and *Lachnospiraceae* NK4A136, **(C)** β-amyloid immunofluorescence in the hippocampus (CA1) and *Clostridium sensu stricto 1*, and **(D)** Iba-1 immunofluorescence in the hippocampus (CA1) and *Clostridium sensu stricto 1*. Spearman’s correlation was used for all analyses (results in box).

To account for within the group, variability correlation analyses were conducted to scrutinize the relationship between the intestinal microbiota and behavior/brain pathology. The analyses revealed that locomotor activity, including distance moved and velocity of movement, positively correlated with putatively beneficial genera *Lachnospiraceae* NK4A136 (Figures 6A,B). In additional, positive correlations were observed between the alcohol-associated genera *Clostridium sensu stricto 1* with β-amyloid and Iba-1 (i.e., microglia) in the hippocampus (Figures 6C,D). These results indicate that *Lachnospiraceae* NK4A136 and *Clostridium sensu stricto 1* may be important mediators of microbiota–brain communication and serve as potential targets for investigation in future studies.

### Impact of alcohol consumption in male mice

#### Chronic alcohol consumption: Blood alcohol and weight gain in male mice

Blood alcohol levels were assessed but were not significant, which is consistent with blood collected following a 2-week withdrawal period (data not shown). Weight gain was also not impacted by genotype nor treatment but was impacted by time (*p* < 0.01), although no interactions were noted (Supplementary Figure S1B). Nonetheless, the changes in the intestinal milieu of alcohol-fed mice are consistent with changes previously observed in alcohol-fed rodents.

#### Chronic alcohol consumption is associated with altered intestinal microbiota in male mice

Stool microbial communities were first examined for a genotype effect, regardless of treatment (i.e., NonTg-H_2_O + NonTg-EtOH vs. 3xTg-AD-H_2_O + 3xTg-AD-EtOH). No significant differences in alpha diversity were observed (data not shown); however, beta diversity was impacted by genotype (Supplementary Figure S3A; Table 1). 3xTg-AD mice had a significantly different microbiota profile, compared to the NonTg mice, which included a concurrent increase in the relative abundance of genera reported to be changed in rodent models and human with neurodegenerative disease including *Parasutterella*, Bacilli RF39, and *Muribaculaceae* (*q* < 0.05) as well as genera that did not meet the stringent *q*-value criterion but were different based on *p*-value (*q* > 0.05; *p* < 0.05) including Lachnospiraceae (genus uncultured), *Lachnospiraceae* ASF356, (all *q* < 0.05), and less stringent *Bifidobacterium* and *Faecalibaculum* (Table 4). Putatively beneficial bacteria that were lower in 3xTg-AD compared to NonTg mice, but it did not meet the stringent criteria for *q-*value, including genera *Ruminococcaceae* UCG-010 and *Lachnospiraceae* FCS020 group (*q* > 0.05; *p* < 0.05)*.* 3xTg-AD male mice also had an increase of the relative abundance of additional genera *Clostridia* (genus unknown) and decrease in the relative abundance of the taxa *Ruminococcus incertae sedis*, *Anaeroplasma*, *Staphylococcus*, *Bilophila*, and Erysipelatoclostridiaceae (genus unknown) (*q* < 0.05) (Table 4). Taken together, these data are consistent with a significantly different microbiota community in the 3xTg-AD male mice compared to the NonTg mice.

**TABLE 4:** DeSeq2—NonTg vs. 3xTg-AD—males. DeSeq2 analysis. Taxa shown have adjusted *p*-values (*p*-value < 0.05 indicated by italics; *q*-value < 0.05 indicated by bold). Base mean = mean of normalized samples. Log2 FC = Log2 fold change of taxa in 3xTg-AD mice in comparison to NonTg mice samples within the respective genotype.

DeSeq2—NonTg vs. 3xTg-AD—males				
Genera (phylum)	Base mean	Log2 FC 3xTg-AD over NonTg	*p*-value	q-value
NonTg (H_2_O + EtOH, *n* = 20) vs. 3xTg-AD (H_2_O + EtOH, *n* = 20)				
Putative pro-inflammatory (*)				
*Parasutterella* (Proteobacteria)	136.62	2.58	*< 0.01*	**0.03**
Bacilli RF39 (Firmicutes)	122.82	1.63	*< 0.01*	**< 0.01**
*Muribaculaceae* (Bacteroidota)	14183.22	0.74	*< 0.01*	**0.02**
Neurodegenerative disease related (**)				
*Bifidobacterium* (Actinobacteriota)	896.02	2.58	*0.01*	0.08
*Faecalibaculum* (Firmicutes)	1060.75	2.48	*0.01*	0.08
Lachnospiraceae (genus uncultured) (Firmicutes)	98.40	1.55	*< 0.01*	**< 0.01**
*Lachnospiraceae* ASF356 (Firmicutes)	30.24	1.36	*0.01*	**0.04**
Putatively beneficial				
*Ruminococcaceae* UCG-005 (Firmicutes)	9.84	−1.72	*0.02*	0.10
*Lachnospiraceae* FCS020 group (Firmicutes)	21.70	−1.05	*0.02*	0.10
Additional genera				
Clostridia (genus unknown) (Firmicutes)	50.94	2.62	*< 0.01*	<0.01
*Erysipelotrichaceae* (Firmicutes)	2.26	2.44	*0.03*	0.10
*Erysipelatoclostridium* (Firmicutes)	81.09	1.66	*0.02*	0.10
*Lachnoclostridium* (Firmicutes)	206.97	−0.48	*0.02*	0.10
*Oscillibacter* (Firmicutes)	191.08	−0.55	*0.04*	0.14
*Alistipes* (Bacteroidota)	399.22	−0.57	*< 0.05*	0.18
*Ruminococcus Incertae Sedis* (Firmicutes)	98.71	−1.21	*< 0.01*	**< 0.01**
*Monoglobus* (Firmicutes)	41.18	−1.73	*0.02*	0.10
*Bacteroides* (Bacteroidota)	377.55	−1.85	*0.02*	0.10
*Streptococcus* (Firmicutes)	16.30	−2.01	*0.02*	0.10
Ruminococcaceae; (*Eubacterium*)*siraeum group* (Firmicutes)	38.36	−2.49	*0.01*	0.05
*Anaeroplasma* (Firmicutes)	23.61	−2.56	*< 0.01*	**0.04**
*Staphylococcus* (Firmicutes)	4.75	−3.53	*< 0.01*	**0.02**
*Bilophila* (Desulfobacterota)	10.21	−3.86	*< 0.01*	**0.01**
Erysipelatoclostridiaceae (genus unknown) (Firmicutes)	8.13	−6.05	*< 0.01*	**< 0.01**

Microbiota was additionally analyzed to understand the impact of alcohol consumption (i.e., regardless of genotype: NonTg-H_2_O + 3xTg-AD-H_2_O vs. NonTg-EtOH + 3xTg-AD-EtOH). No significant differences in alpha diversity indices were noted (data not shown), but analysis of beta diversity revealed significant differences in the microbial community (Supplementary Figure S3B; Table 1). Alcohol treatment was associated with a decrease in Bacilli (genus unknown) and *Candidatus Arthromitus* (*q* < 0.05) (Table 5). In additional, alcohol consumption was associated with changes in other bacterial genera based on a less stringent threshold for significance (*q* > 0.05; *p* < 0.05) including genera *Clostridium sensu stricto 1* and *Ruminococcus*, along with lower relative abundance of beneficial SCFA-producing genera *Lachnospiraceae* UCG-006, *Lachnospiraceae* NK4B4, *Clostridia* vadin BB60, and *Erysipelatoclostridium* (Table 5). Taken together alcohol treatment was associated with changes that are consistent with those reported in the literature.

**TABLE 5:** DeSeq2—H_2_O vs. EtOH—males. DeSeq2 analysis. Adjusted *p*-values (*p*-value < 0.05 indicated by italics; *q*-value < 0.05 indicated by bold). Base mean = mean of normalized samples. Log2 FC = Log2 fold change of taxa in EtOH-fed mice in comparison to H_2_O-fed mice samples within the respective genotype.

DeSeq2—H_2_O vs. EtOH—males				
Genera (Phylum)	Base mean	Log2 FC EtOH over H_2_O	*p*-value	q-value
H_2_O (NonTg + 3xTg-AD, *n* = 20) vs. EtOH (NonTg + 3xTg-AD, *n* = 20)				
Alcohol consumption implicated				
*Clostridium sensu stricto 1* (Firmicutes)	395.40	2.15	*0.02*	0.29
*Ruminococcus* (Firmicutes)	155.81	1.65	*0.01*	0.29
Putatively beneficial (*)				
*Lachnospiraceae* NK4B4 group (Firmicutes)	2.93	−2.28	*0.03*	0.32
*Lachnospiraceae* UCG-006 (Firmicutes)	111.14	−1.12	*0.04*	0.32
Additional genera				
*Ruminococcaceae* UBA 1819 (Firmicutes)	2.76	2.46	*0.01*	0.29
*Dorea* (Firmicutes)	10.95	1.86	*0.04*	0.32
*Monoglobus* (Firmicutes)	41.18	1.54	*0.04*	0.32
*Oscillospirales* UCG-010 (Firmicutes)	17.51	1.34	*0.02*	0.29
Anaerovoracaceae; (*Eubacterium*) *nodatum group* (Firmicutes)	16.33	1.16	*0.02*	0.29
Ruminococcaceae*; Incertae Sedis* (Firmicutes)	98.71	0.74	*0.03*	0.32
*Clostridia* vadin BB60 group (Firmicutes)	87.35	−0.77	*0.04*	0.32
*Erysipelatoclostridium* (Firmicutes)	81.09	−1.69	*0.02*	0.29
*Candidatus Arthromitus* (Firmicutes)	1.41	−3.84	*< 0.01*	**0.04**
Bacilli (genus unknown) (Firmicutes)	513.66	−4.30	*< 0.01*	**< 0.01**
NonTg: H_2_O (*n* = 10) vs. EtOH (*n* = 10)				
Alcohol consumption implicated (*)				
*Bifidobacterium* (Actinobacteriota)	896.02	8.17	*< 0.01*	**< 0.01**
*Dubosiella* (Firmicutes)	1383.89	6.70	*< 0.01*	**< 0.01**
*Faecalibaculum* (Firmicutes)	1060.75	5.70	*< 0.01*	**< 0.01**
*Romboutsia* (Firmicutes)	145.24	3.73	*0.02*	0.18
*Prevotellaceae* UCG-001 (Bacteriodota)	765.86	3.35	*0.01*	0.10
*Clostridium sensu stricto 1* (Firmicutes)	395.4	3.11	*0.03*	0.28
*Turicibacter* (Firmicutes)	1085.91	2.69	*0.04*	0.32
Putatively Beneficial				
*Marvinbryantia* (Firmicutes)	78.3	−2.54	*0.04*	0.35
Additional genera				
*Peptococcus* (Firmicutes)	3.76	6.28	*< 0.01*	**< 0.01**
Atopobiaceae (genus unclassified) (Actinobacteriota)	78.89	4.80	*< 0.01*	**< 0.05**
*Monoglobus* (Firmicutes)	41.18	2.29	*0.04*	0.32
*Oscillospirales* UCG-010 (Firmicutes)	17.51	2.19	*0.01*	0.10
Anaerovoracaceae; (*Eubacterium*) *nodatum group* (Firmicutes)	16.33	2.02	*< 0.01*	0.08
*Candidatus Arthromitus* (Firmicutes)	1.41	−3.99	*0.05*	0.35
Bacilli (genus unclassified) (Firmicutes)	513.66	−6.44	*< 0.01*	**< 0.01**
3xTg-AD:H_2_O (*n* = 10) vs. EtOH (*n* = 10)				
Alcohol consumption implicated				
*Ruminococcus* (Firmicutes)	81.09	1.79	*0.03*	0.63
Putatively Beneficial				
*Lachnospiraceae* GCA-900066575 (Firmicutes)	1.5	−1.51	*0.02*	0.63
*Erysipelatoclostridium* (Firmicutes)	228.31	−2.11	*0.03*	0.63
*Lachnospiraceae* UCG-004 (Firmicutes)	513.66	−2.89	*0.01*	0.36
Additional genera				
Lachnospiraceae; (*Eubacterium*) *xylanophilum group* (Firmicutes)	76.2	1.51	*0.02*	0.63
Ruminococcaceae*; Incertae Sedis* (Firmicutes)	145.5	1.32	*0.05*	0.68
*Colidextribacter* (Firmicutes)	155.81	0.52	*0.04*	0.68
Bacilli (genus unknown) (Firmicutes)	98.71	−2.87	*< 0.01*	0.30

Microbial communities were subsequently reviewed for treatment effects within each genotype. In NonTg mice, alcohol consumption did not impact alpha diversity (data not shown) but did significantly influence beta diversity (Supplementary Figure S3C; Table 1). Specifically, alcohol consumption was associated with increased relative abundance of multiple chronic alcohol consumption–implicated genera *Faecalibaculum*, *Bifidobacterium*, and *Dubosiella* (all *q* < 0.05) and less stringent *Clostridium sensu stricto 1*, *Turicibacter*, *Romboutsia*, and *Prevotellaceae* UCG-001 (all *q* > 0.05; *p* < 0.05). The NonTg alcohol–consuming male mice additionally had a decrease in the relative abundances of SCFA-producing bacteria *Marvinbryantia* (*q* < 0.05) (Table 5). Additional bacterial changes include an increase in genera *Peptococcus* and Atopobiaceae (genus unclassified) with a concurrent decrease in Bacilli (genus unclassified) (all *q* < 0.05) (Table 5).

In 3xTg-AD mice, there were no significant effects observed for alpha diversity (data not shown). However, alcohol consumption was associated with a significant change in beta diversity (Supplementary Figure S3D; Table 1). Although an overall difference was noted in the microbial communities, no taxa reached the stringent level of significance set in this study (Table 5, *q* < 0.05). However, bacteria were different using a less stringent criterion (*p* < 0.05) including higher relative abundance of chronic alcohol consumption–associated genus *Ruminococcus*, with lower relative abundance beneficial SCFA-producing genera *Lachnospiraceae* UCG-004, *Lachnospiraceae* GCA-900066575, and *Erysipelatoclostridium* and additional Bacilli (genus unclassified) in the alcohol-consuming mice (Table 5).

Taken together, the observed richness within the intestinal microbiota was not impacted by genotype or treatment; however, microbial communities in samples from each treatment group were more similar to each other as compared to microbial communities in other groups. We observed that 3xTg-AD male mice have a different microbiota composition then NonTg male mice (genotype effect), but that alcohol impacts the microbial communities in both 3xTg-AD and NonTg male mice (alcohol treatment effect).

#### Chronic alcohol consumption is associated with disrupted intestinal barrier integrity in male mice

**FIGURE 7:**
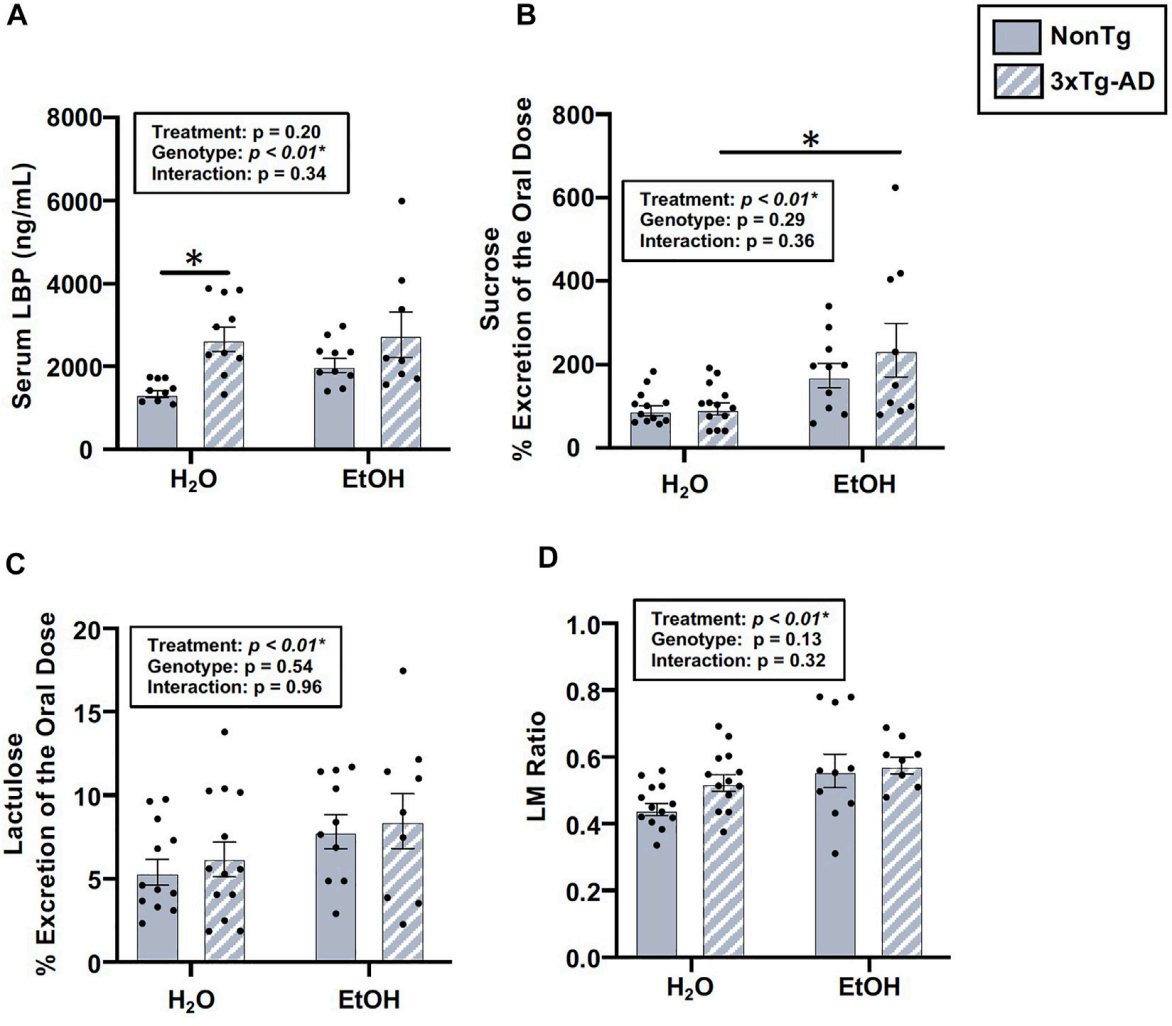
Effect of alcohol consumption on intestinal barrier integrity in male mice. **(A)** Serum LBP exhibited a significant effect of genotype, but it did not exhibit a significant impact of alcohol treatment nor an interaction. **(B)** Urinary sucrose did not exhibit a significant effect of the genotype (or interaction), but it was impacted by alcohol treatment. **(C)** Urinary lactulose did not exhibit a significant effect of the genotype (nor an interaction), but it was impacted by alcohol treatment. **(D)** Urinary lactulose:mannitol (LM) ratio was not impacted by the genotype (nor was there an interaction), but it exhibited a significant effect of alcohol treatment. Between *n* = 6–10 mice/treatment group. Two-way ANOVA (results in box) was followed by planned (i.e., *a priori*) between-group comparisons, which are indicated on each graph when significant: ^∗^ *p* < 0.05.

The intestinal barrier, assessed *via* urinary sugar content, did not identify a significant main effect of genotype, but genotype was a significant factor for LBP (Figure 7A). Between-group comparisons revealed that LBP levels were higher in H_2_O-fed 3xTg-AD mice compared to H_2_O-fed NonTg mice (Figure 7A). While genotype only impacted LBP, alcohol treatment impacted multiple assessments of intestinal barrier integrity, including sucrose, lactulose, and LM ratio (Figures 7B–D), but no main effect of alcohol treatment was observed for mannitol or sucralose (data not shown). Between-group comparisons demonstrated that urinary levels of sucrose were higher in alcohol consuming compared to water-fed 3xTg-AD mice (Figure 7B). Alcohol treatment did not significantly impact serum LBP levels in male mice (Figure 7A). Taken together, these data are consistent with alcohol consumption disrupting intestinal barrier integrity. These data are summarized in Supplementary Table S2.

#### Chronic alcohol consumption did not alter AD-relevant behavior or brain pathology in male mice

**FIGURE 8:**
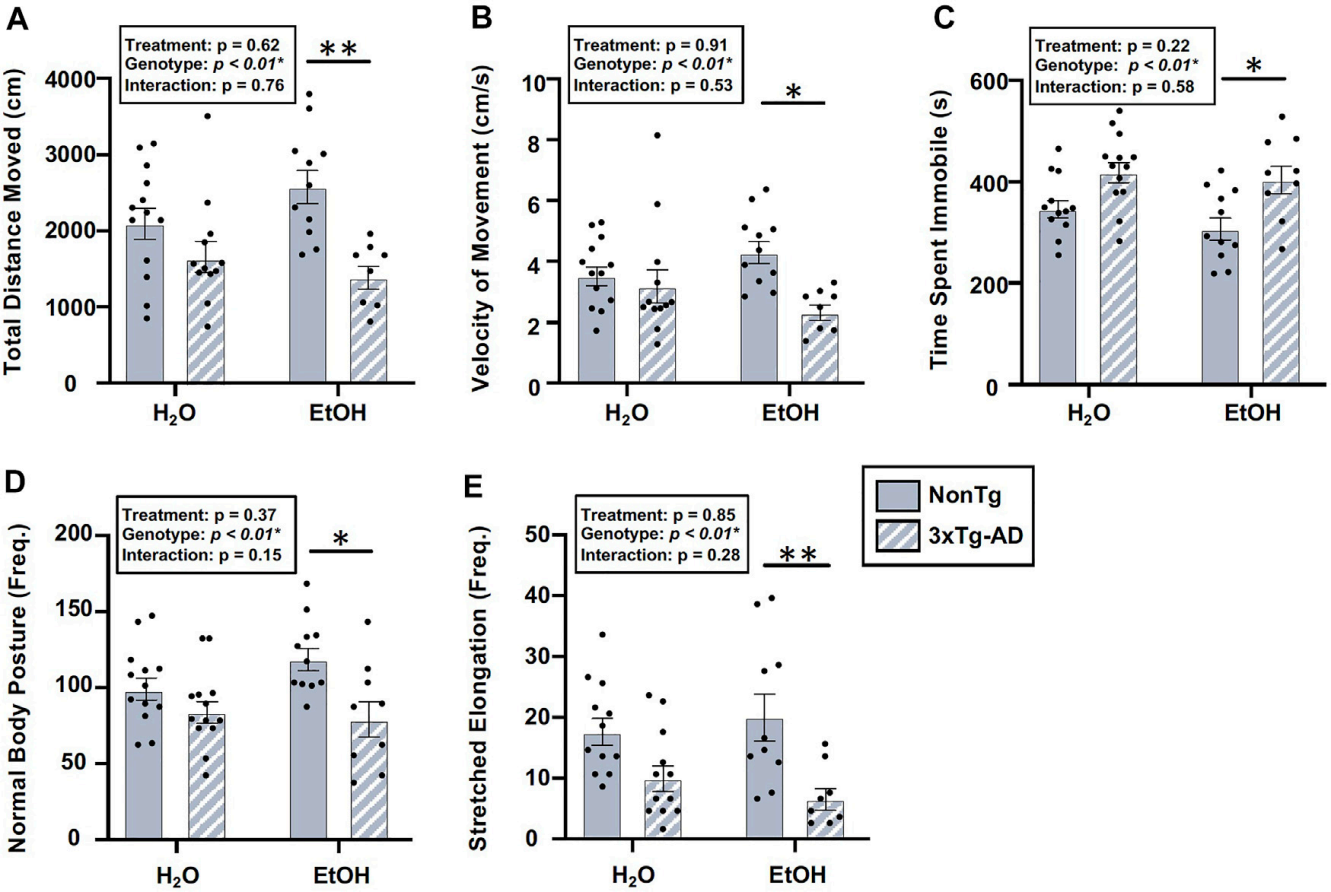
Effect of alcohol consumption on behavior in male mice. **(A)** Total distance moved was significantly impacted by the genotype, but it did not exhibit a significant effect of alcohol treatment nor was there an interaction. **(B)** Velocity of movement was significantly impacted by the genotype but not by alcohol treatment nor was there an interaction. **(C)** Time spent immobile was significantly impacted by the genotype, but it was not impacted by alcohol treatment nor was there an interaction. **(D)** Frequency of normal body posture was impacted by the genotype but not by alcohol treatment nor was there an interaction. **(E)** Frequency of stretched elongation body posture was significantly impacted by the genotype but not alcohol treatment nor was there an interaction. Between *n* = 6–10/treatment group. Two-way ANOVA (results in box) was followed by planned (i.e., *a priori*) between-group comparisons, which are indicated on each graph when significant: ^∗^ *p* < 0.05 and ^∗^^∗^ *p* < 0.01.

There was a significant effect of genotype in several behaviors assessed using the OFT, including total distance moved, velocity of movement, time spent immobile, frequency of normal body posture, and frequency of stretched elongation posture (Figure 8), but not time spent in center (data not shown). Between-group comparisons revealed significant differences between alcohol-consuming NonTg and 3xTg-AD mice in which 3xTg-AD mice tend to have less movement; their movements are slower, with a concurrent increase in time spent immobile (Figure 8). The analysis did not reveal a significant effect of alcohol treatment on any behavior assessed.

**FIGURE 9:**
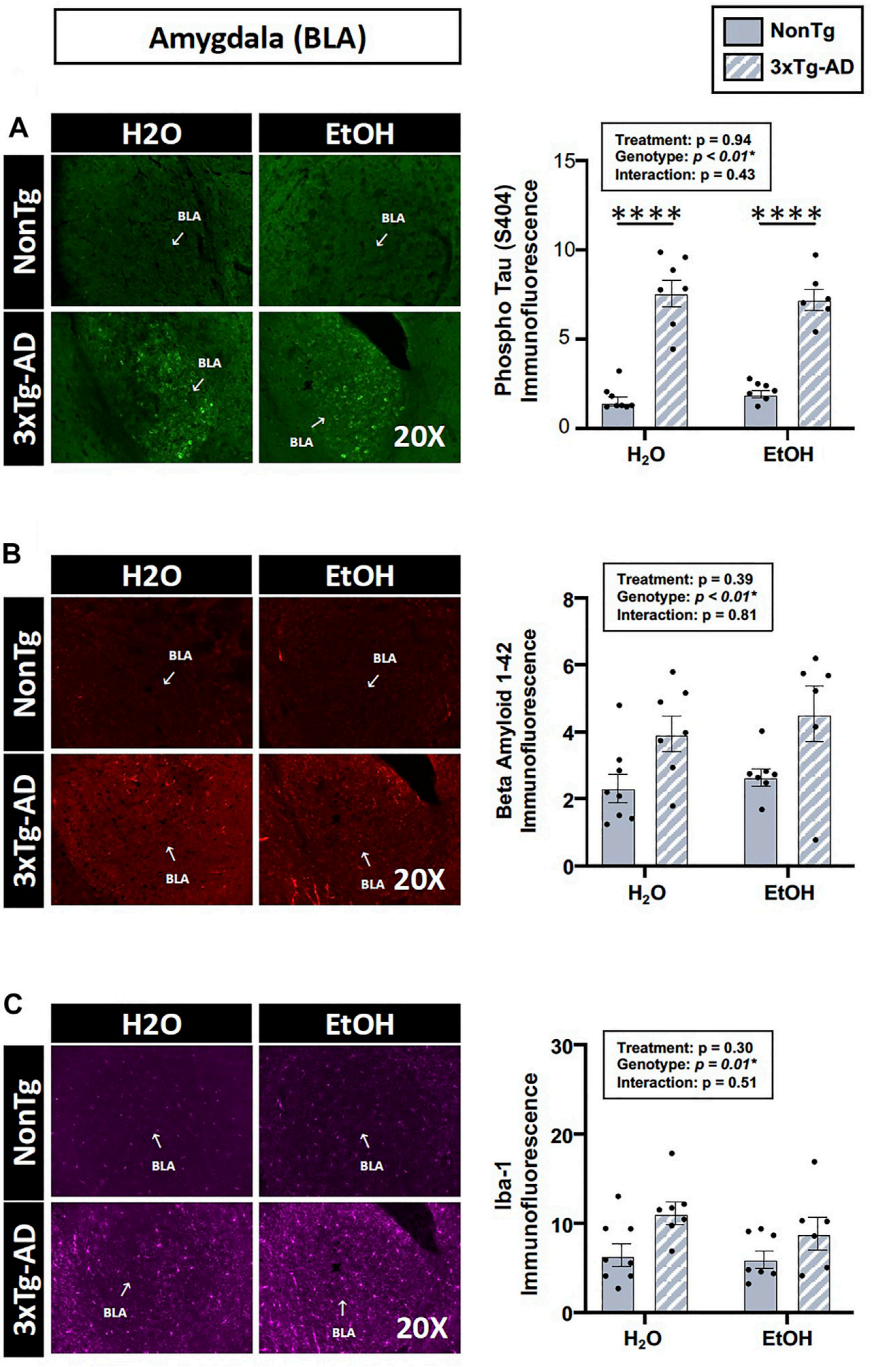
Effect of alcohol consumption on AD-like brain outcomes in male mice. Basal lateral amygdala (BLA). **(A)** Phosphorylated tau exhibited a significant effect of genotype but was not affected by alcohol treatment nor was there an interaction. **(B)** β-amyloid showed a significant effect of genotype but was not affected by alcohol treatment nor was there an interaction. **(C)** Iba-1 demonstrated a significant effect of genotype but was not affected by alcohol treatment nor was there an interaction. Between *n* = 6–10 mice/treatment group. Two-way ANOVA (results in box) was followed by planned (i.e., *a priori*) between-group comparisons, which are indicated on each graph when significant: ^∗^^∗^^∗^^∗^ *p* < 0.0001.

The analysis of brain tissue indicated a significant main effect of genotype for phospho tau, β-amyloid, and Iba-1 immunofluorescence in the BLA (Figure 9), but no effects of genotype were noted in the hippocampus (data not shown). Between-group testing revealed that phospho tau was higher in 3xTg-AD mice compared to NonTg mice in both H_2_O and EtOH-fed mice (Figure 9). No main effects of alcohol treatment on brain pathology outcomes were noted in BLA or hippocampus (Figure 9). These data are summarized in Supplementary Table S2.

#### Alcohol consumption did not alter peripheral inflammation in male mice

**FIGURE 10:**
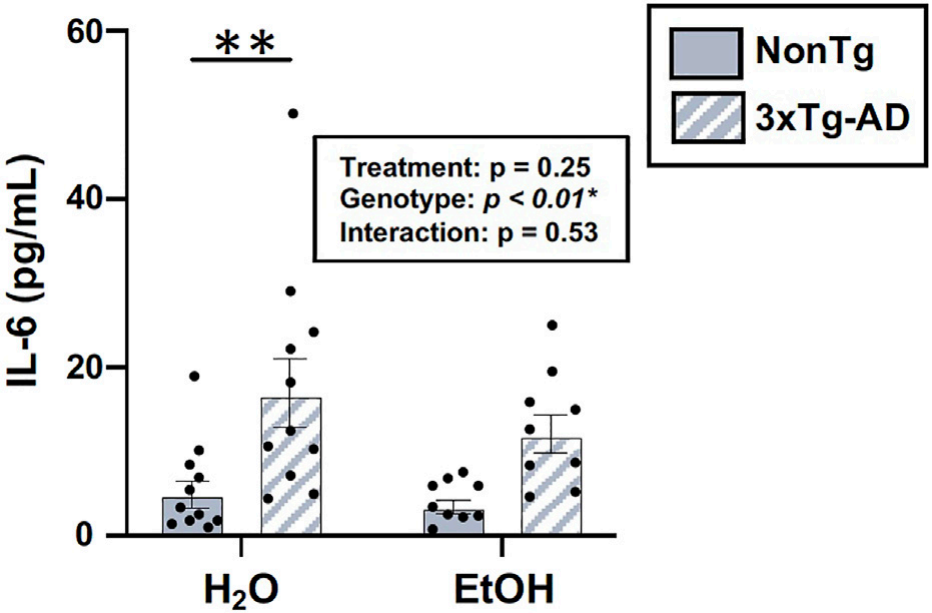
Effect of alcohol consumption on peripheral inflammation in male mice. Serum cytokine IL-6 levels were assessed. IL-6 (pg/ml) was impacted by the genotype but not by alcohol treatment nor was there an interaction. Between *n* = 6–14 mice/treatment group. Two-way ANOVA (results in box) was followed by planned (i.e., *a priori*) between-group comparisons, which are indicated on each graph when significant: ^∗^^∗^ *p* < 0.01.

One way that the intestinal microbiota and the intestinal barrier can impact the brain is by altering inflammation; therefore, serum IL-6 was examined. An analysis revealed a significant effect of genotype, but there was no treatment effect nor was there an interaction (Figure 10). The lack of alcohol treatment–associated peripheral inflammation could account for why alcohol did not potentiate the AD-like phenotype in 3xTg-AD mice. A summary of these data is found in Supplementary Table S2.

#### Relationship between the microbiota and AD-relevant outcomes in male mice

A correlation analysis was conducted to assess the relationship between the relative abundances of species taxa and AD-relevant outcomes. No significant relevant relationships were observed between the intestinal microbiota and behaviors or brain staining pathology (data not shown).

#### Females vs. males

Males and females had distinct intestinal microbiomes which prompted separate analyses of sex. To further understand and characterize sex-specific differences in response to alcohol, additional analyses were conducted. Sex-specific differences were noted for barrier integrity (i.e., sucrose, LM ratio), behavior (all behaviors except time spent in center), and brain pathology (Iba-1, β-amyloid) (Supplementary Table S3). However, no interactions (sex x alcohol treatment interaction) were noted for any outcome meaning sex did not impact the response to alcohol. Nonetheless, the sex differences in conjunction with the significant correlations between the intestinal microbiota and AD-relevant outcomes that were exclusively observed in females (Figure 6) are intriguing and suggest that males and females are distinct and should be analyzed separately.

## Discussion

Although alcohol consumption caused microbiota dysbiosis and intestinal barrier dysfunction (consistent with studies by our group and others) (Keshavarzian et al., 2009; Mutlu et al., 2009, 2012; Summa et al., 2013; Patel et al., 2015; Bishehsari et al., 2017; Swanson et al., 2020; Lee and Lee, 2021), these changes were not sufficient to exacerbate the behavioral phenotype or AD-like brain pathology in 3xTg-AD mice. The lack of an impact of alcohol consumption on the AD-like phenotype is surprising since previous studies have demonstrated that alcohol promotes cognitive dysfunction and exacerbates brain pathology in rodent models of AD that persist at least as long as 1-month after alcohol consumption has ceased (Hoffman et al., 2019; Gong et al., 2021). For example, administration of 25% alcohol (16 weeks) to 3xTg-AD mice impairs cognition and is associated with increased phospho tau (Ser199/202) burden compared to non-alcohol–consuming 3xTg-AD mice (Hoffman et al., 2019) and administration of 4% alcohol to APP/PS1 double transgenic AD mice increases β-amyloid (Gong et al., 2021). This begs the question: why did alcohol not exacerbate AD-like behavior and brain pathology in this study? It is possible that the alcohol-induced changes in the microbiota do not mediate the impact of microbiota on the brain (i.e., alcohol can directly impact the brain). However, there is an evergrowing body of literature demonstrating that the intestinal microbiota can influence the brain (Luczynski et al., 2016; Sherwin et al., 2018; Ma et al., 2019; Millman et al., 2021).

One possibility accounting for the discrepancy between the current and prior studies could be differences in microbial communities observed between laboratories (so called “cage effects”). Baseline microbial community can affect microbiome response to alcohol (Lavelle et al., 2019; Rashidi et al., 2021) and it is possible that the baseline differences in microbial communities between institutions may contribute to differences observed between studies. In additional, in this study, differences were noted in microbial communities in NonTg and 3xTg-AD mice and this may have impacted the observed response to alcohol. For example, differences in microbial communities at baseline may contribute to a ceiling effect which is supported by a lack of interaction (genotype x treatment) observed in this study. In fact, it is intriguing to consider that the abnormal microbiota observed in the 3xTg-AD mice may contribute to the behavioral abnormalities and brain pathology in 3xTg-AD mice, but this assertion will require additional investigation.

An additional reason why alcohol did not exacerbate AD-like behavior and brain pathology in this study could be the lack of systemic inflammation (i.e., IL-6). Although alcohol changed the microbiota and influenced the intestinal barrier, this was not associated with an increase in IL-6. There are many different mechanisms by which the microbiota and intestinal barrier can communicate with the brain (immunity, metabolites, extracellular vesicles), but the outcomes from this study suggest that peripheral inflammation may be important. Additional studies will be required to determine if peripheral inflammation is an important mechanism contributing to microbiota–brain axis communication in 3xTg-AD mice.

In additional, several experimental factors may have contributed to the lack of impact of alcohol consumption on AD-like behavior and pathology in this study. The first issue to address is age. Alcohol treatment was initiated when mice were 10 weeks of age and continued until 30 weeks of age. It is possible that initiating alcohol treatment at a younger or older age may influence outcomes as age-associated changes in intestinal barrier function are noted (Man et al., 2015) including in 3xTg-AD mice (Chen et al., 2020). Alcohol treatment duration may also be important. It is possible that a treatment duration of longer than 20 weeks is necessary to observe an effect. Finally, alcohol dose is also an important consideration. In this study 20% alcohol was administered based on data from our group indicating this is sufficient to induce microbiota dysbiosis and intestinal barrier dysfunction, but a higher dose of alcohol may be necessary to impact systemic inflammation and behavior/brain pathology in 3xTg-AD mice. Future studies investigating age, alcohol treatment duration, and alcohol dose are necessary to better understand the relationship of alcohol and the gut–brain axis in 3xTg-AD mice.

There are some limitations associated with this study that may have contributed to lack of alcohol-induced effects in this study. 1) Mice were group housed in this study, and consequently the amount alcohol consumed by each individual mouse was not assessed. Future studies should carefully monitor food/alcohol intake and collect blood during alcohol treatment to assess blood alcohol levels which will aid in interpretation of the outcomes. 2) The OFT was used to assess anxiety, motor behavior, and other non-memory associated AD-like behaviors in this study. Although other studies have used the OFT to assess behavioral abnormalities in rodent models of AD (Bryan et al., 2009; Hebda-Bauer et al., 2013) future studies should assess the impact of alcohol on tests focused on cognition and memory (e.g., novel object recognition or Morris water maze).

Despite the lack of an impact of alcohol consumption on behavior and brain pathology, important information was revealed in this study. There were genotype effects noted in microbial communities between NonTg and 3xTg-AD mice. The cause of these genotype differences (e.g., immune function, intestinal barrier, intestinal motility) and the biological impact of these differences are important to consider. Perhaps the microbiota differences observed in 3xTg-AD mice contribute to the phenotype but investigating this possibility will require further studies. In additional, outcomes assessed in females and males were not identical in this study. For example, females exhibited significant correlations between microbiota and behavior and brain pathology while males did not, and sex was a factor that significantly impacted intestinal barrier integrity, behavior, and brain pathology. Sex-dependent differences in microbiota have already been noted in rodents and humans and sex differences may (at least in part) explain why women are more susceptible to the detrimental effects of alcohol (e.g., liver disease) (Cox et al., 2019; Kim et al., 2020; Peng et al., 2020; Kaur et al., 2021).

Although alcohol consumption did not promote the AD-like phenotype, the abnormal microbiota in 3xTg-AD may contribute to the AD-like phenotype additional investigations are needed to fully interpret outcomes. However, intriguing results suggest that the microbiota may influence the development of the AD-like phenotype in 3xTg-AD mice and sex differences may be important. A better understanding of the microbiota–brain axis in 3xTg-AD mice and AD in general may be an opportunity to influence disease course and identify novel therapeutic targets.

## Data Availability

The datasets presented in this study can be found in online repositories. The names of the repository/repositories and accession number(s) can be found at: https://www.ncbi.nlm.nih.gov/, PRJNA781947.
