# Supplementary material for: Impact of alcohol-induced intestinal microbiota dysbiosis in a rodent model of Alzheimer’s disease
Source: Front Aging. 2022 Aug 15;3:916336. doi: 10.3389/fragi.2022.916336 (PMC9421609; doi:10.3389/fragi.2022.916336)
Supplement: Supplementary file 1 [file DataSheet2.pdf]

Supplemental Table

Supplemental Table 1. Shannon Index, Simpson's Index, observed features (richness), and Pielou's evenness were measured at the features (ASV) level. Datasets were rarefied to 6,500 sequences per sample. Mean index score and standard deviation (SD) displayed. (p-value < 0.05, indicated by italics). Analysis: Mann-Whitney U Test (non-parametric data based on Shapiro Wilk test). All groups were combined for each sex. NonTg H2O-fed (n=10); NonTg EtOH-fed (n=10); 3xTg-AD H2O-fed (n=10); 3xTg-AD EtOH-fed (n=10)

Supplemental Table 2. Summary table for all two-way ANOVA analyses.

Supplemental Table 3. Male vs. Female Outcome Difference. Two-way ANOVA analysis (factors: sex, alcohol treatment, interaction). Significance was p-value < 0.05 (indicated by italics).

Supplemental Figures

Supplemental Figure 1. Chronic alcohol consumption and weight in female and male mice. Data for each week of treatment (x-axis) is represented by difference in body weight, measured as the percent difference from baseline (y-axis). **(A)** Female mice. Weight exhibited a significant effect of time but was from individual factors treatment or genotype. However, there was a significant treatment x genotype x time interaction. **(B)** Male mice. Weight exhibited a significant effect of time, but was not impacted by treatment or genotype nor was there an interaction between any of the factors. Between n = 6-14 mice / treatment group. Three-way ANOVA (results in box).

Supplemental Figure 2. Intestinal microbiota community profiles in females. Principal coordinate analysis (PCoA) was used to visualize the data and PERMANOVA statistics are indicated on each plot. Stacked column plots depicting the average relative abundance of bacterial phyla of the different treatment groups are shown with microbiota taxa that were significantly different between groups (assessed by DeSeq2 (p-value < 0.05)) indicated in bold. **(A)** NonTg (H2O and EtOH) versus 3xTg-AD (H2O and EtOH). Communities were significantly different between groups (q < 0.01). **(B)** H2O-fed (NonTg and 3xTg-AD) versus EtOH-fed

(NonTg and 3xTg-AD genotypes grouped). Communities were significantly different between groups ($q < 0.01$). **(C)** H2O-fed NonTg versus EtOH-fed NonTg mice. Communities were not significantly different between groups ($q = 0.07$). **(D)** H2O-fed 3xTg-AD versus EtOH-fed 3xTg AD mice. Communities were significantly different between groups ($q < 0.01$).

Supplemental Figure 3. Intestinal microbiota community profiles in males. Principal coordinate analysis (PCoA) was used to visualize the data and PERMANOVA statistics are indicated on each plot. Stacked column plots depicting the average relative abundance of bacterial phyla of the different treatment groups are shown with microbiota taxa that were significantly different between groups (assessed by DeSeq2 (p-value < 0.05)) indicated in bold. **(A)** NonTg (H2O and EtOH) versus 3xTg-AD (H2O and EtOH). Communities were significantly different between groups ($q < 0.01$). **(B)** H2O-fed (NonTg and 3xTg-AD) versus EtOH-fed (NonTg and 3xTg-AD). Communities were significantly different between groups ($q < 0.01$). **(C)** H2O-fed NonTg versus EtOH-fed NonTg. Communities were not significantly different between groups ($q > 0.01$). **(D)** H2O-fed 3xTg-AD versus EtOH-fed 3xTg-AD mice. Communities were significantly different between groups ($q > 0.01$).
