# Supplementary figures and images for: Impact of alcohol-induced intestinal microbiota dysbiosis in a rodent model of Alzheimer’s disease
Source: Front Aging. 2022 Aug 15;3:916336. doi: 10.3389/fragi.2022.916336 (PMC9421609; doi:10.3389/fragi.2022.916336)

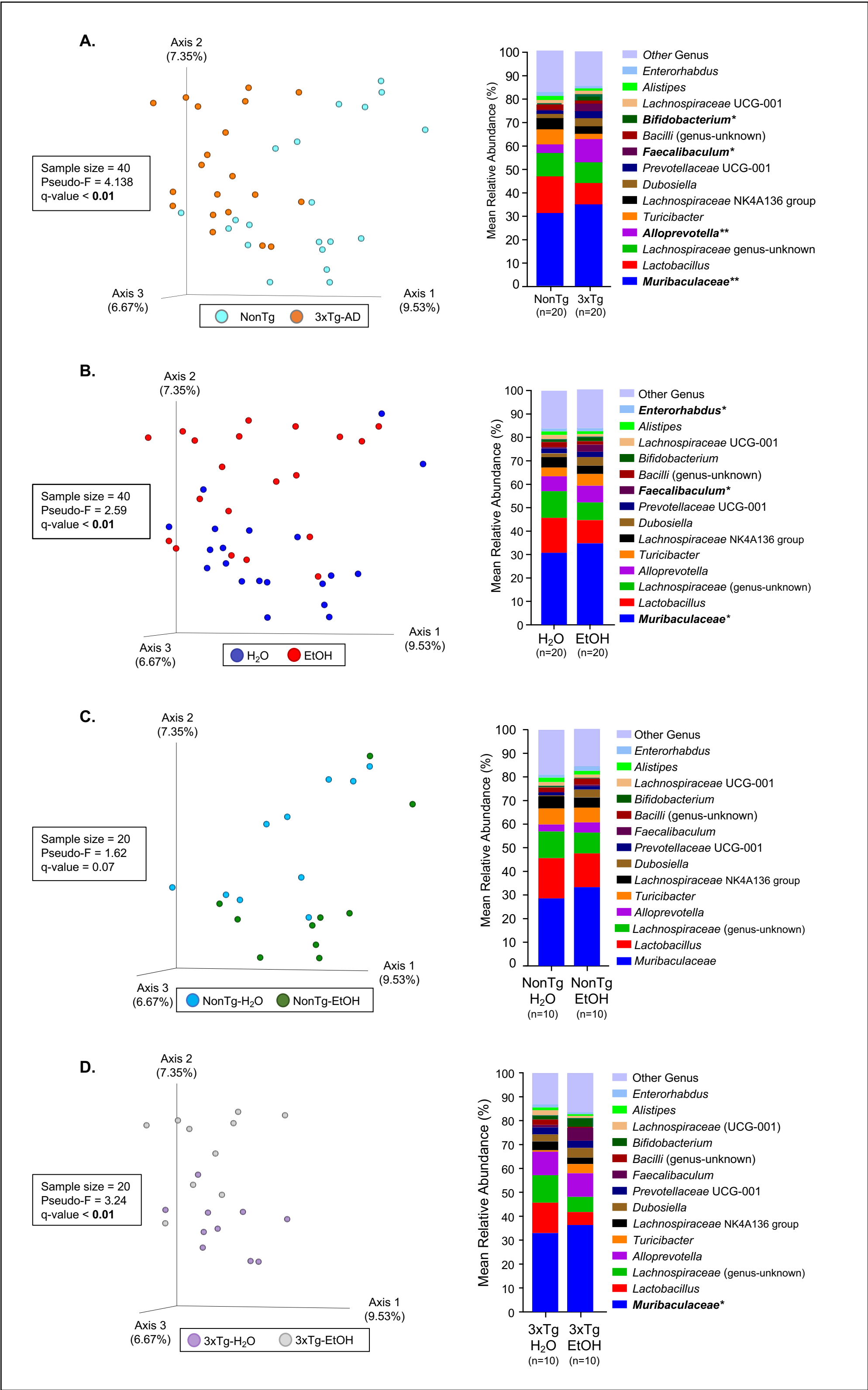

Supplement: Supplementary file 2 [file Image2.PDF]

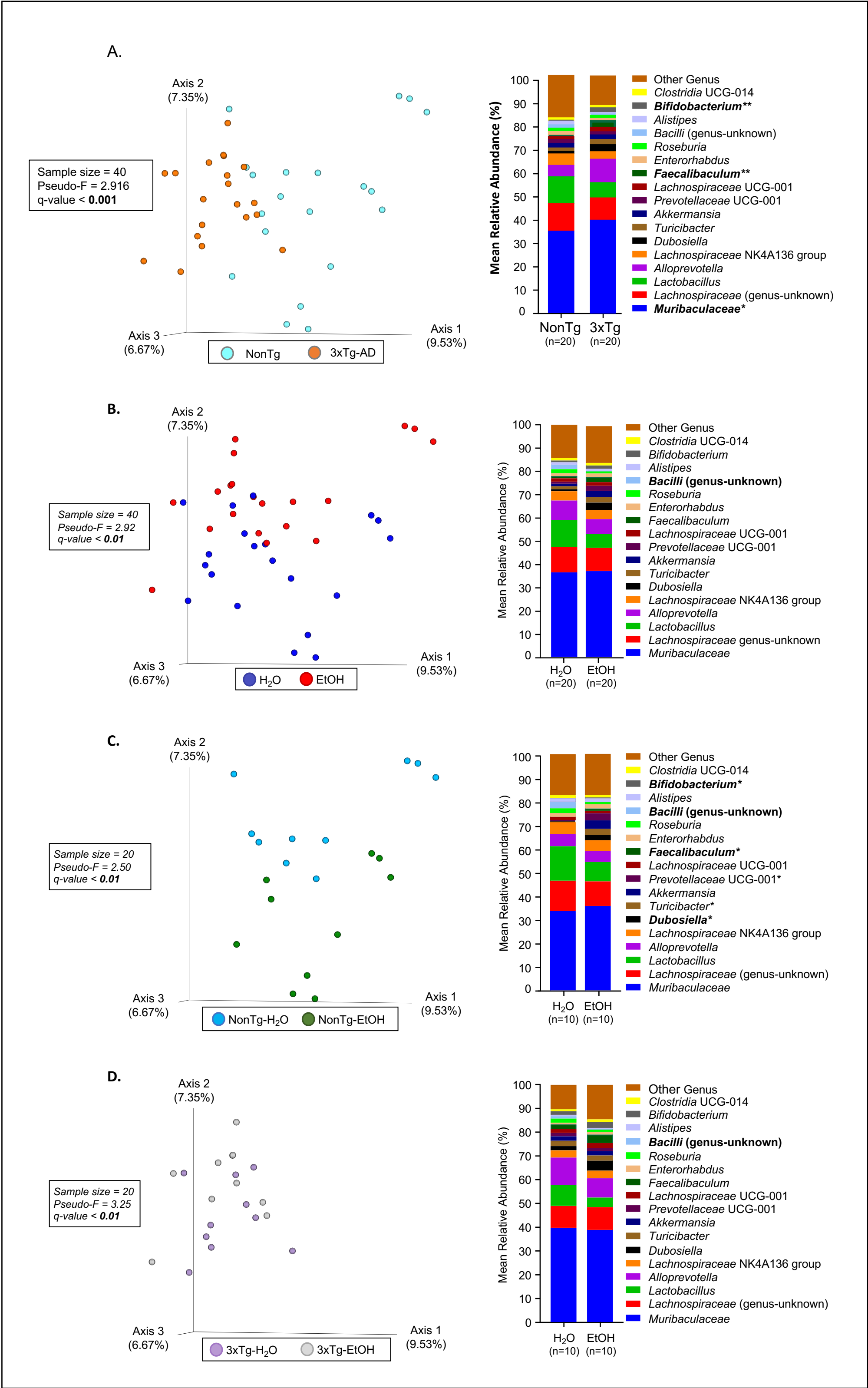

Supplement: Supplementary file 3 [file Image3.PDF]

A.

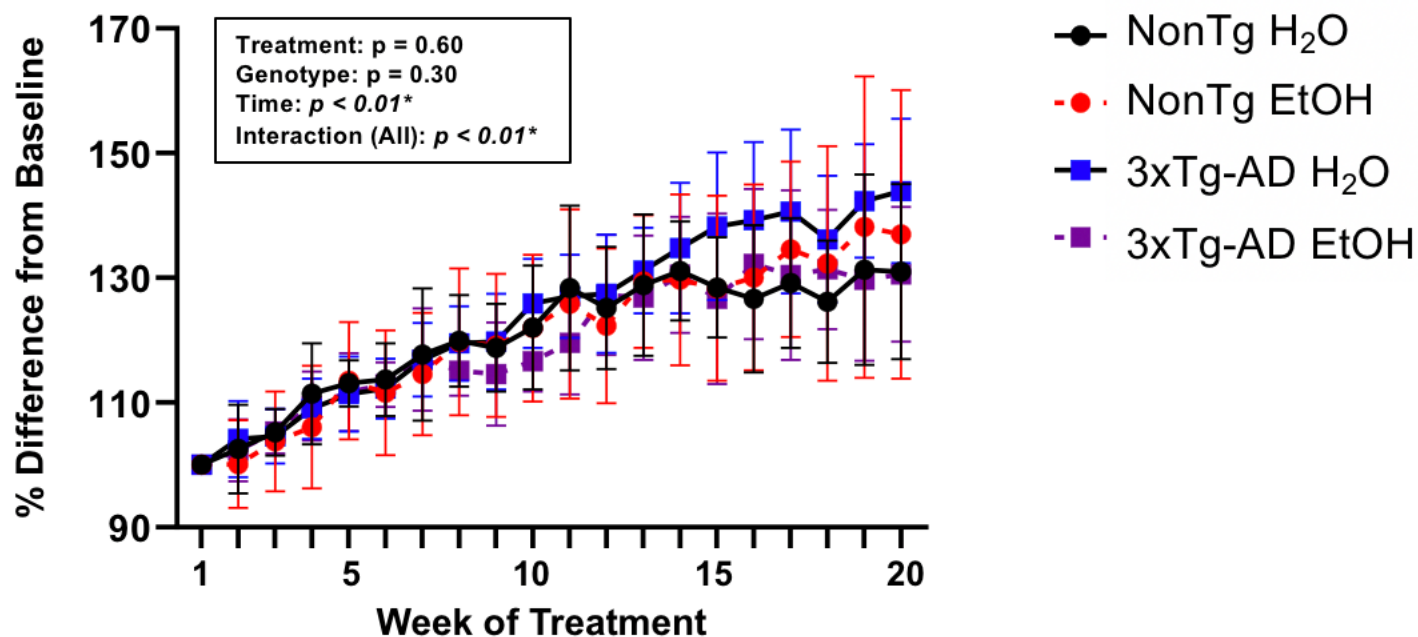

B.

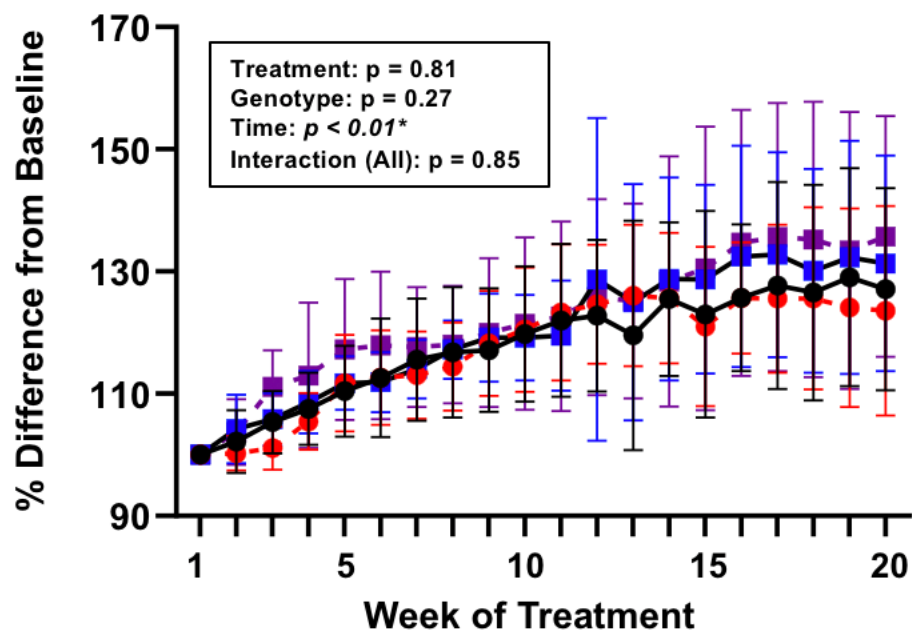

Supplement: Supplementary file 5 [file Image1.pdf]
