# Supplementary material for: Impact of alcohol-induced intestinal microbiota dysbiosis in a rodent model of Alzheimer’s disease
Source: Front Aging. 2022 Aug 15;3:916336. doi: 10.3389/fragi.2022.916336 (PMC9421609; doi:10.3389/fragi.2022.916336)
Supplement: Supplementary file 4 [file DataSheet1.docx]

| **Supplemental Table 1. Sex Differences in Alpha-Diversity.** | | | |
| --- | --- | --- | --- |
|  | **Females**  **Mean ± SD** | **Males**  **Mean ± SD** | **p-value** |
| Shannon Index | 5.81 ± 0.56 | 6.10 ± 0.46 | *<0.01* |
| Simpson Index | 0.96 ± 0.02 | 0.97 ± 0.02 | *<0.01* |
| Richness | 206.50 ± 45.31 | 222.50 ± 42.01 | *0.01* |
| Evenness | 0.76 ± 0.05 | 0.79 ± 0.04 | *<0.01* |

| **Supplemental Table 2. Two-way ANOVA Outcomes Summary.** | | | | | |
| --- | --- | --- | --- | --- | --- |
|  | | **Sex** | **Treatment** | **Genotype** | **Interaction** |
| **Intestinal Barrier Integrity** | | | | | |
| Mannitol | | Female | *0.03* | *0.048* | 0.98 |
|  |  | Male | 0.34 | 0.87 | 0.92 |
| Sucrose | | Female | *< 0.01* | < *0.01* | 0.08 |
|  |  | Male | *< 0.01* | 0.29 | 0.36 |
| Lactulose | | Female | *< 0.01* | <*0.01* | 0.59 |
|  |  | Male | *< 0.01* | 0.54 | 0.96 |
| Sucralose | | Female | *0.03* | 0.87 | 0.68 |
|  |  | Male | 0.17 | 0.57 | 0.86 |
| Lactulose:Mannitol (LM) Ratio | | Female | 0.10 | *0.01* | 0.18 |
|  |  | Male | *< 0.01* | 0.13 | 0.32 |
| LBP | | Female | *0.01* | *0.03* | 0.36 |
|  |  | Male | 0.20 | *< 0.01* | 0.34 |
| **Peripheral Inflammation** | | | | | |
| IL-6 | | Female | 0.85 | 0.08 | 0.09 |
|  |  | Male | 0.25 | *0.01* | 0.53 |
| **Behavior (OFT)** | | | | | |
| Total Distance Moved (cm) | | Female | 0.19 | 0.45 | 0.83 |
|  |  | Male | 0.62 | *< 0.01* | 0.08 |
| Velocity of Movement (cm/s) | | Female | 0.18 | 0.46 | 0.82 |
|  |  | Male | 0.91 | *< 0.01* | 0.053 |
| Time Spent in Center (s) | | Female | 0.68 | *0.02* | 0.17 |
|  |  | Male | 0.69 | 0.73 | 0.62 |
| Stretched Elongation Posture (Freq.) | | Female | 0.30 | *< 0.01* | 0.79 |
|  |  | Male | 0.85 | *< 0.01* | 0.28 |
| Normal Body Posture (Freq.) | | Female | *< 0.01* | *< 0.01* | 0.83 |
|  |  | Male | 0.37 | *< 0.01* | 0.15 |
| Time Spent Immobile (s) | | Female | 0.25 | 0.95 | 0.85 |
|  |  | Male | 0.22 | *< 0.01* | 0.58 |
| **Brain Pathology** | | | | | |
| Amygdala | Tau | Female | 0.86 | *< 0.01* | 0.80 |
|  |  | Male | 0.94 | *< 0.01* | 0.43 |
|  | β-Amyloid | Female | 0.84 | *< 0.01* | 0.16 |
|  |  | Male | 0.39 | *< 0.01* | 0.92 |
|  | Iba-1 | Female | 0.41 | *0.01* | 0.10 |
|  |  | Male | 0.30 | *0.01* | 0.51 |
| Hippocampus | Tau | Female | 0.12 | 0.07 | 0.07 |
|  |  | Male | 0.40 | 0.24 | 0.92 |
|  | β-Amyloid | Female | 0.83 | *0.01* | 0.52 |
|  |  | Male | 0.70 | 0.31 | 0.98 |
|  | Iba-1 | Female | 0.99 | *0.01* | 0.79 |
|  |  | Male | 0.31 | 0.68 | 0.87 |

| **Supplemental Table 3. Male vs. Female Outcome Difference.** | | | | | | | |
| --- | --- | --- | --- | --- | --- | --- | --- |
|  | | **NonTg**  Female-H_2_0 (n=10), Female-EtOH (n=10), Male-H_2_0 (n=10), Male-EtOH (n=10) | | | **3xTg-AD**  Female-H_2_0 (n=10), Female-EtOH (n=10), Male-H_2_0 (n=10), Male-EtOH (n=10) | | |
|  |  | Interaction | Alcohol Treatment | Sex | Interaction | Alcohol Treatment | Sex |
| **Intestinal Barrier integrity** | | | | | | | |
| Mannitol | | 0.66 | 0.07 | 0.25 | 0.68 | 0.20 | 0.38 |
| Sucrose | | 0.13 | *<0.01* | *<0.01* | 0.36 | *<0.01* | 0.24 |
| Lactulose | | 0.83 | *0.01* | 0.06 | 0.76 | *0.02* | 0.86 |
| Sucralose | | 0.75 | *0.01* | 0.10 | 0.67 | 0.21 | 0.42 |
| Lactulose:Mannitol (LM) Ratio | | 0.06 | *0.04* | *0.04* | 0.77 | *0.02* | 0.06 |
| LBP | | 0.82 | *0.01* | 0.07 | 0.12 | 0.07 | 0.37 |
| **Behavior (OFT)** | | | | | | | |
| Total Distance Moved (cm) | | 0.07 | 0.78 | 0.40 | 0.96 | 0.24 | *<0.01* |
| Velocity of Movement (cm/s) | | 0.07 | 0.80 | 0.39 | 0.61 | 0.14 | *0.01* |
| Time Spent in Center (s) | | 0.45 | 0.39 | 0.14 | 0.95 | 0.36 | 0.31 |
| Normal Body Posture (Freq.) | | *0.01* | 0.92 | 0.08 | 0.44 | 0.20 | *0.02* |
| Stretched Elongation Posture (Freq.) | | 0.38 | 0.83 | *0.04* | 0.79 | 0.16 | 0.49 |
| Time Spent Immobile (s) | | 0.15 | 0.85 | 0.51 | 0.39 | 0.88 | *<0.01* |
| **Brain Pathology** | | | | | | | |
| CA1 | Tau | 0.54 | 0.69 | 0.45 | 0.29 | *0.049* | *0.03* |
|  | β-Amyloid | 0.83 | 0.51 | 0.74 | 0.63 | 0.75 | *<0.01* |
|  | Iba1 | 0.43 | 0.59 | *<0.01* | 0.62 | 0.50 | 0.40 |
| BLA | Tau | 0.97 | 0.25 | 0.16 | 0.92 | 0.86 | 0.30 |
|  | β-Amyloid | 0.60 | 0.22 | 0.17 | 0.30 | 0.98 | 0.19 |
|  | Iba1 | 0.55 | 0.88 | 0.29 | 0.92 | 0.053 | 0.08 |
